# Detectability and cortical depth dependence of stimulus-driven high-frequency BOLD oscillations in the human primary somatosensory and motor cortex

**DOI:** 10.1162/imag_a_00427

**Published:** 2025-01-13

**Authors:** Shota Hodono, Jonathan R. Polimeni, David Reutens, Martijn A. Cloos

**Affiliations:** Donders Centre for Cognitive Neuroimaging, Donders Institute for Brain, Cognition and Behaviour, Radboud University, Nijmegen, Netherlands; Centre for Advanced Imaging, The University of Queensland, Brisbane, QLD, Australia; Athinoula A. Martinos Center for Biomedical Imaging, Department of Radiology, Harvard Medical School, Massachusetts General Hospital, Charlestown, MA, United States; Harvard–MIT Program in Health Sciences and Technology, MIT, Cambridge, MA, United States; ARC Training Centre for Innovation in Biomedical Imaging Technology (CIBIT), The University of Queensland, Brisbane, QLD, Australia

**Keywords:** fMRI, BOLD, hemodynamics, vascular hierarchy, temporal specificity

## Abstract

In functional magnetic resonance imaging (fMRI), neural activity is inferred from the associated hemodynamic response. However, the degree to which hemodynamics can track dynamic changes in neuronal activity, and thus the ultimate temporal resolution of fMRI, remains unknown. To evaluate the detectability of stimulus-driven high-frequency blood oxygenation level dependent (BOLD) signal oscillations in functionally and vascularly distinct cerebral cortical areas, stimuli up to 0.5 Hz were used to evoke activation in the primary somatosensory and motor cortex. Despite their functional and vascular differences, a similar frequency dependence was observed in both cortical areas. We then proceeded to investigate these signals at different levels of the cortical vascular hierarchy, using cortical depth as a proxy. We observed that, above 0.33 Hz, the BOLD response amplitude decreased faster with increasing frequency near the pial surface than in the parenchyma, suggesting that, in addition to exhibiting high spatial specificity, parenchymal signals—accessible with high spatial resolution imaging—also attenuate less rapidly when the stimulus frequency is increased. In addition, as the stimulus frequency increased, we observed larger relative phase differences in the BOLD oscillations across cortical depths. When averaged across depths, these signals can thus interfere destructively, suggesting that high spatial resolutions can avoid this phase cancellation and thereby aid in the detection of rapid BOLD oscillations.

## 1 Introduction

Functional magnetic resonance imaging (fMRI) is one of the most widely used methods to noninvasively study brain activity and functional connectivity (Bandettini, 2007;Heeger & Ress, 2002;Logothetis, 2008). Modern MRI systems allow the acquisition of high spatial resolution fMRI data with subsecond sampling time and whole-brain coverage (Griswold et al., 2002;Moeller et al., 2010;Pruessmann et al., 1999;Sodickson & Manning, 1997;Uğurbil, 2014;Wiggins et al., 2006). However, all fMRI techniques currently in use infer neural activation from the associated hemodynamic changes, which leads to a blurred localization of neuronal activity in both space and time (Hillman, 2014;Turner, 2002).

The hemodynamic response—and thus the spatial specificity of fMRI—is strongly influenced by the vascular architecture (Kim & Ugurbil, 2003;Turner, 2002;Zhao et al., 2004). The cerebral cortex has a hierarchical vascular organization, consisting of large feeding arteries on the surface, from which intracortical arterioles descend into the cortex, where they divide into arteriolar branches, then feed the capillary networks of the parenchyma, which are in turn drained by venular branches and ascending venules, which finally collect into large draining veins on the surface (Duvernoy et al., 1981).

In particular, the most commonly used fMRI signal, the gradient-echo blood oxygenation level dependent (BOLD) contrast (Ogawa et al., 1990), is dominated by changes from large veins (Kim & Ugurbil, 2003;Turner, 2002;Zhao et al., 2004). Therefore, the BOLD response varies systematically with cortical depth (Fracasso et al., 2018;Kashyap et al., 2018;Koopmans et al., 2010;Marquardt et al., 2018;Polimeni et al., 2010); typically, it is strongest around large draining veins near the pial surface that collect blood from extended domains of tissue, which are often downstream from the site of neural activation, giving it poor spatial specificity. Moving progressively deeper into the cortex, smaller and more spatially specific BOLD responses can be found from the capillaries and small venular branches within the parenchyma (Polimeni et al., 2010).

The relationship between the vascular hierarchy and the temporal specificity of the BOLD response is less well understood (Polimeni & Lewis, 2021). Although the canonical hemodynamic response function (HRF) (Friston et al., 1994)—which is used in conventional fMRI analysis to summarize the response of the vasculature to neuronal activity—predicts vanishingly small BOLD signal changes in response to neural oscillations above ~0.1 Hz,Lewis et al. (2016)showed that visual stimulus-driven neuronal oscillations at frequencies as high as 0.75 Hz still produce observable rapid BOLD oscillations at these same frequencies in human primary visual cortex (V1). Since then, evidence for high-frequency BOLD oscillations has been observed in human auditory cortex (Frühholz et al., 2020) and in rat somatosensory cortex (Cao et al., 2019). This suggests that high-frequency BOLD oscillations may be observable broadly across the brain and in different mammals. However, it remains unknown how such high-frequency stimulus-driven BOLD responses are shaped by—and vary within—the local vascular architecture.

Brief stimuli combined with a long interstimulus interval (ISI) are often used to study the transient dynamics of the BOLD response (Boynton et al., 1996;Glover, 1999;Yeşilyurt et al., 2008). Such short-duration stimuli have a broad temporal frequency bandwidth enabling straightforward estimation of a BOLD “impulse” response—provided that the ISI is sufficiently long to allow the BOLD response to fully return to baseline—that does not require any assumption of linear superposition of overlapping responses. However, these experiments do not directly address whether high-frequency periodic neuronal activation can be detected in the corresponding BOLD response, particularly when the stimulus is repeated sufficiently rapidly such that the BOLD response does not return to baseline between trials. Following the lead ofLewis et al. (2016), we used stimuli and ISIs of equal durations to drive the BOLD response into a steady state to study the detectability of stimulus-driven BOLD oscillations at specific frequencies.

To extend our understanding of the biophysical constraints that limit the ultimate resolution of BOLD fMRI in space and time, we performed fMRI using a task-based paradigm designed to evoke steady-state BOLD signal oscillations at specific frequencies from 0.05 Hz up to 0.5 Hz. We tailored the task to target human primary somatosensory (S1) and motor (M1) cortices because they have distinct neuronal and vascular architectures (Duvernoy et al., 1981;Palomero-Gallagher & Zilles, 2019). In adult humans, the primary somatosensory cortex (S1) is granular and is one of the thinnest cerebral cortical areas with a thickness of about 2 mm, whereas the primary motor cortex (M1) is agranular (Palomero-Gallagher & Zilles, 2019;Yamawaki et al., 2014) and one of the thickest cortical areas with a thickness close to 4 mm (Fischl & Dale, 2000). From a hemodynamic point of view, cortical thickness determines, in part, the distance blood travels across layers on its way into and out of the cortex and, therefore, should influence the time taken for blood to travel from the parenchyma up to the large draining veins on the surface (Turner, 2002). Furthermore, the differences in myelo- and cytoarchitecture of these two areas are likely to reflect differences in angio-architecture such as microvascular density, and indeed capillary distributions in these two areas have been demonstrated to be strikingly different (Blinder et al., 2013;Duvernoy et al., 1981). Thus, studying M1 and S1 allowed us to investigate the detectability of stimulus-driven high-frequency BOLD signal oscillations at different levels of the cortical vascular hierarchy—using cerebral cortical depth as a proxy (Polimeni et al., 2010;Siero et al., 2011)—in two architecturally distinct areas and test whether high temporal specificity of the BOLD response may be a general feature present throughout the vascular hierarchy in a variety of cortical areas.

## 2 Methods

### 2.1 Subjects

Nine healthy volunteers (23–35 years old, mean 28.4 years; two male, seven female, seven right-handed based on Edinburgh Handedness Inventory (Oldfield, 1971), having provided prior written informed consent, were scanned at 7 Tesla (Magnetom, Siemens, Germany) using a 32-channel head coil (Nova Medical, USA). One of nine subjects was eliminated from the analysis due to significant motion confounds. The study was approved by the local human research ethics committee in accordance with national guidelines.

### 2.2 Hand motion capture

During each fMRI session, the subjects wore a custom-built dataglove to record their hand kinematics and monitor task performance in real time (Supplementary Fig. 1a) (Hodono et al., 2023). In addition, the glove also provided tactile stimulation as the fingertips rub against the inner fabric of the glove. The dataglove encoded proximal interphalangeal joint angles of the left hand with a sampling frequency of 15 Hz. An increase in joint angle indicates flexion; a decrease in joint angle indicates extension. The motion ranged from fully closed to the relaxed position. In brief, the dataglove was built around a fire-retardant glove (OMP, Ronco Scrivia, Italy). Cotton pockets were stitched across the proximal interphalangeal joints, each holding a piezoresistive flex-sensor (SKU: SEN-1026). The sensors connected to a custom interface board, holding fuses (TR5 372, 100 mA) and a nonmagnetic RJ45 socket. The interface board was mounted in a 3D-printed housing held on the back of the glove using Velcro. A Cat5/6 ethernet cable was used to carry the signals out of the magnet room through a custom-built filter panel. The other side of the filter was connected to an Arduino ZERO equipped with a shielded custom interface board. Each pair of twisted wires connected a single sensor to an ADC to quantify the joint angle by measuring the change in resistance in the sensor. A Teensy 3.6 circuit board was used to split the trigger signal from the MR system into two separate paths (Supplementary Fig. 1b). One was connected to the Arduino ZERO interface board to synchronize recorded hand motion data with MR images (Supplementary Fig. 1c). The other was connected to the trigger box used to synchronize the visual cues. All of the data were sent to a computer through a serial connection for real-time monitoring and storage.

Recorded hand measurements were retrospectively synchronized to MRI data time series. Detected hand motion blocks were defined by thresholding the first-order derivatives (using a Savitzky-Golay filter) of the joint angle with respect to time. The number of flexions within each on-block was estimated by thresholding their first-order derivatives. Then, the grasping frequency was estimated from the detected on-block duration and the number of flexions.

### 2.3 fMRI task

First, a functional localizer experiment was performed to define M1 and S1 ROIs. A block paradigm with 20 s “on” and “off” (0.025 Hz) was used. Within “on” blocks, subjects performed the ~2 Hz flexion–extension for the full block duration. During off-blocks they held their fingers in a comfortable resting position (Supplementary Fig. 2). The z-score map was computed from the localizer experiment using general linear model analysis implemented in FSL (https://fsl.fmrib.ox.ac.uk/fsl/) with the default double-gamma HRF (Woolrich et al., 2001). Subsequently, a series of “fast fMRI” experiments was performed to elucidate the range of observable stimulus-driven signal oscillations in M1 and S1. In these experiments, the subjects were instructed only to move the fingers of their left hand (wearing the dataglove). On- and off-block durations were varied to evoke neuronal oscillations at 0.05, 0.10, 0.20, 0.33, and 0.50 Hz (Supplementary Fig. 2,Supplementary Movies 1–4). To increase SNR, experiments targeting higher frequencies were repeated multiple times (Supplementary Tables 1 and 2). The total scan time per subject needed to cover all frequencies exceeding 2 hours. Therefore, each subject participated in multiple scan sessions targeting different frequency ranges (Supplementary Table 2). Each session acquired data using a 0.05- and 0.1-Hz stimulus for reference, in addition to acquiring a functional localizer and anatomical reference. A custom-written program (C++, OpenGL) was used to display visual cues indicating the start and end of each block and provide a rhythm for the periodic flexing motion. Both dataglove recordings and visual cues were synchronized with the MRI scanner. In addition, we also collected fMRI data without task to estimate the noise contribution to the data. During these “no-task” runs, the subjects were asked to maintain a relaxed position and refrain from moving. One subject was invited to participate in another session to evaluate noise properties in the cortical depth analysis. In the session, the no-task run was repeated for 10 times while visual cues for the 0.5-Hz task were presented. From here on, we will refer to these tasks by their intended stimulus frequency, or as no-task.

### 2.4 Canonical BOLD hemodynamic response function (HRF) predictions

Our experiments entailed hand movements initiated by cueing the subject in a boxcar-timing design. Such a stimulus contains multiple harmonic frequencies in its spectrum, which may complicate analysis. Therefore, we first modeled the expected BOLD response through convolution with the canonical HRF from the SPM toolbox (https://www.fil.ion.ucl.ac.uk/spm/), based on both the idealized paradigm timing (visual cues) and the actual subject performance (recorded hand motion) (Hodono et al., 2023). The results were Fourier transformed and their spectra were analyzed.

### 2.5 Data acquisition

To help with slice prescription, a 1.0-mm isotropic resolution T1-weighted dataset was collected using a 3D MP2RAGE with TR = 4300 ms, TE = 2.35 ms, flip angle 1 = 5°, flip angle 2 = 6°, FOV in readout = 265 mm, 256 × 256 matrix, 176 slices, slice partial Fourier = 6/8, GRAPPA factor = 3, bandwidth = 250 Hz/px, echo spacing = 6.4 ms (Supplementary Fig. 3a). Distortion-matched anatomical reference images with 1.1-mm isotropic resolution were obtained using a T1-EPI sequence (Renvall et al., 2016) with TR = 5 s, TE = 25 ms, flip angle = 90°, 42 slices, no gap, 143-mm × 143-mm FOV with 1.1-mm isotropic resolution, no partial Fourier, GRAPPA factor = 3, nominal echo spacing 0.82 ms, 43 measurements with a slice permutation factor of 1 between consecutive inversion recovery periods. Synthetic MPRAGE images were created from the estimated quantitative T1 maps (Supplementary Fig. 3b). The same EPI readout, matching the T1-EPI, was used to obtain all functional images. The CMRR 2D GRE-EPI sequence (Moeller et al., 2010) was used with TR = 0.5 s, TE = 24.6 ms, flip angle = 40°, 18 slices, 143-mm × 143-mm FOV with 1.1-mm isotropic resolution, no partial Fourier, GRAPPA factor = 3, multiband factor = 2 with FOV/2 CAIPI shift, nominal echo spacing 0.82 ms, 490 measurements including 10 initial dummy scans. The tSNR maps are seen inSupplementary Figure 4. An example of the slice positioning is presented inSupplementary Figure 3a. For eight out of nine subjects, a 20-s off block was added to the beginning of each run (total of 530 measurements). Total number of runs performed per session per subject can be seen inSupplementary Table 2.

### 2.6 Preprocessing

All functional data were corrected for slice timing and motion using SPM12. No spatial filtering was applied. The estimated motion parameters were analyzed in the frequency domain to evaluate potential motion confounds. By design, our anatomical reference image has the same geometric distortion as the functional images; therefore, no distortion corrections were applied. M1 and S1 on the contralateral/right hemispheres (stimulated side) and two control ROIs were drawn manually using FSLeyes from the FSL package based on the activation map acquired from the functional localizer run (Supplementary Figs. 5 and 6). The two control ROIs were drawn within the cortical gray matter in anterior regions of both hemispheres away from the region of activation (Supplementary Fig. 5). To maintain similar levels of statistical power between the ROIs, the volume of control ROIs was adjusted to match that of the corresponding M1 ROI for each subject. The number of voxels included in the S1 and M1 ROIs is reported inSupplementary Table 3. Rigid-body coregistration of all functional data to the same-session anatomical reference T1-EPI data was performed using ANTs (Advanced Normalization Tools) (Avants et al., 2011) and ITK-SNAP (Yushkevich et al., 2006). A linear temporal detrending was used to account for signal drift on a voxel-by-voxel basis.

### 2.7 Steady-state BOLD response analysis

The steady-state response was isolated by removing the transient response at the beginning of each measurement run. To this end, all data collected before the start of the trial closest to the 65-s mark were removed. The BOLD response analysis was performed in each subject’s native space, without smoothing. The steady-state BOLD responses were analyzed in both the frequency and time domains. Because each session contained a dedicated distortion-matched anatomical reference and a dedicated functional localizer, there was no need for coregistration across sessions; instead, each scan session has its own dedicated set of ROIs (Supplementary Table 3).

#### 2.7.1 Frequency domain analysis

The steady-state BOLD response time series was Fourier transformed and averaged across voxels within each given ROI in the frequency domain. For each subject, the estimated amplitude spectra of the BOLD response to different stimulus frequencies were normalized to the amplitude spectrum of the 0.05-Hz stimulus data collected within the same session: the spectra from the M1 and control ROIs were all normalized to the reference 0.05-Hz stimulus data from the M1 ROI, and the spectra from the S1 ROI were normalized to the reference 0.05-Hz stimulus data from the S1 ROI. The normalized amplitude spectra from the M1 and S1 ROIs were then averaged across subjects and plotted on a semilogarithmic scale.

For comparison, we also processed the “no-task” run obtained for each subject to estimate the noise. Assuming that the spectral amplitude at each frequency represented an independent noise estimate, we averaged these amplitude spectra across all voxels within the M1 ROI. The resulting mean amplitude spectra were then averaged over subjects to estimate average noise floor in our experiments. The noise floors for experiments performed using different number of runs were then estimated by dividing the single run noise floor by the square root of the number of runs.

#### 2.7.2 Time domain analysis

The mean trial–response was estimated by partitioning the time series into individual trials based on the detected hand motion onset and averaged to visualize percentage signal change of the stimulus-driven oscillating BOLD response. Here, one “trial” consisted of one “on” and “off” block, and the mean trial response was first calculated voxel by voxel and run by run, then averaged across runs and voxels within the ROI. The 95% confidence intervals were calculated across voxels within the ROI for each time point in the trial.

The BOLD responses were then averaged across subjects using an ROI-based approach in which the trial-averaged responses calculated from each subject were simply averaged together in the time domain, and the frequency spectra of the measured responses were averaged together in the frequency domain; no spatial normalization or cross-subject alignment was performed.

### 2.8 Cortical depth-dependent analysis

The image volumes containing the mean trial responses for each individual subject were upsampled four times in all spatial directions using ANTs (Avants et al., 2011). No explicit spatial smoothing was applied. The ROIs were redrawn on the 4x upsampled T1-EPI-based synthetic MPRAGE images, using the original ROIs defined on the 1.1-mm data as a guide. Cortical depth-dependent ROIs were estimated using equivolume sampling with LayNii (L. R. Huber et al., 2021). Cortical depths were divided in 7 and 15 bins for S1 and M1, respectively, accounting for the approximately twofold difference in cortical thickness between S1 and M1 (Fischl & Dale, 2000). Each of the estimated cortical depth-dependent ROIs can be seen inSupplementary Figure 6, and cortical thickness measured in each subject is reported inSupplementary Table 4.

#### 2.8.1 Response amplitude analysis

To estimate the impact of the vascular hierarchy on the detectability of high-frequency stimulus-driven BOLD oscillations, we calculated the BOLD response amplitude as a function of cortical depth. To increase the SNR, the BOLD responses were averaged across subjects. For comparison, we also processed the 10 “no-task” runs obtained for subject 4 in a similar way to investigate the noise as function of cortical depth.

#### 2.8.2 Response delay analysis

To estimate the response time differences and corresponding relative temporal phase differences across cortical depths, the trial-averaged responses were upsampled 50 times in the time domain to achieve a 10-ms time step. To first order, the trial-averaged response resembles a sinusoid. Therefore, a sinusoid was fitted to estimate the temporal phase difference across cortical depth and stimulus frequency. These steps were performed for each depth independently, without implicit assumptions regarding neuronal response shape or timing between layers in the precentral and postcentral gyrus. To better visualize relative change across cortical depths, the delay profiles were normalized to the delay found in voxels intersecting the CSF in the sulcal space between the M1 and S1 ROIs. This CSF space was chosen as a reference because the vasculature drains toward the pial surface, thus the delay at the pial surface is expected to be longer than that of the parenchyma, and because it is expected that the large veins in this area will produce the highest GRE-BOLD contrast-to-noise ratio (Polimeni et al., 2010). The resulting delay profiles were then averaged over subjects.

#### 2.8.3 Signal cancellation analysis

It is well known that the evolution of the hemodynamic response across the vascular hierarchy smooths the BOLD response in space and time. We hypothesized that, in cases where the temporal frequency is high enough to create large relative phase variations across cortical depths, stimulus-driven BOLD response oscillations occurring at different locations can also interfere destructively with each other, resulting in cancellation in the oscillating BOLD responses. To investigate this hypothesis, we calculated BOLD response amplitudes averaged across individual cortical depths and ROI using

$$\text{Depth Average Signal Change}=\frac{1}{D}\sum_{d=1}^{D}f\left(S_{d}\right)$$ [1]

and

$$\text{ROI Average Signal Change}=f\left(\frac{1}{D}\sum_{d=1}^{D}S_{d}\right),$$ [2]

whereDis the number of cortical depths in the ROI,frepresents the operation of computing the amplitude of the steady-state BOLD response, and$S_{d}$is mean trial-averaged response at depth d. SinceEq. [1]computes the BOLD response amplitude at each depth then calculates the mean, the relative temporal phase of the response at each depth is lost. In contrast,Eq. [2]computes the BOLD response amplitude of the mean of the responses across depths, therefore, BOLD responses having different temporal phases can destructively interfere with each other. Signal cancellation due to destructive interference (or phase cancellation) can, therefore, be estimated as the relative difference between these two quantities, that is, as

$$\text{signal cancellation}=\frac{\left(\text{Depth Average Signal Change}\right)-\left(\text{ROI Average Signal Change}\right)}{\text{Depth Average Signal Change}}$$ [3]

and this signal cancellation measure can be quantified as a function of stimulus frequency to confirm whether the steady-state BOLD response delays across cortical depth cause more cancellation at some stimulus frequencies than others. These measures were then averaged across subjects to summarize the results.

## 3 Results

### 3.1 Canonical HRF predictions

**Fig. 1.**
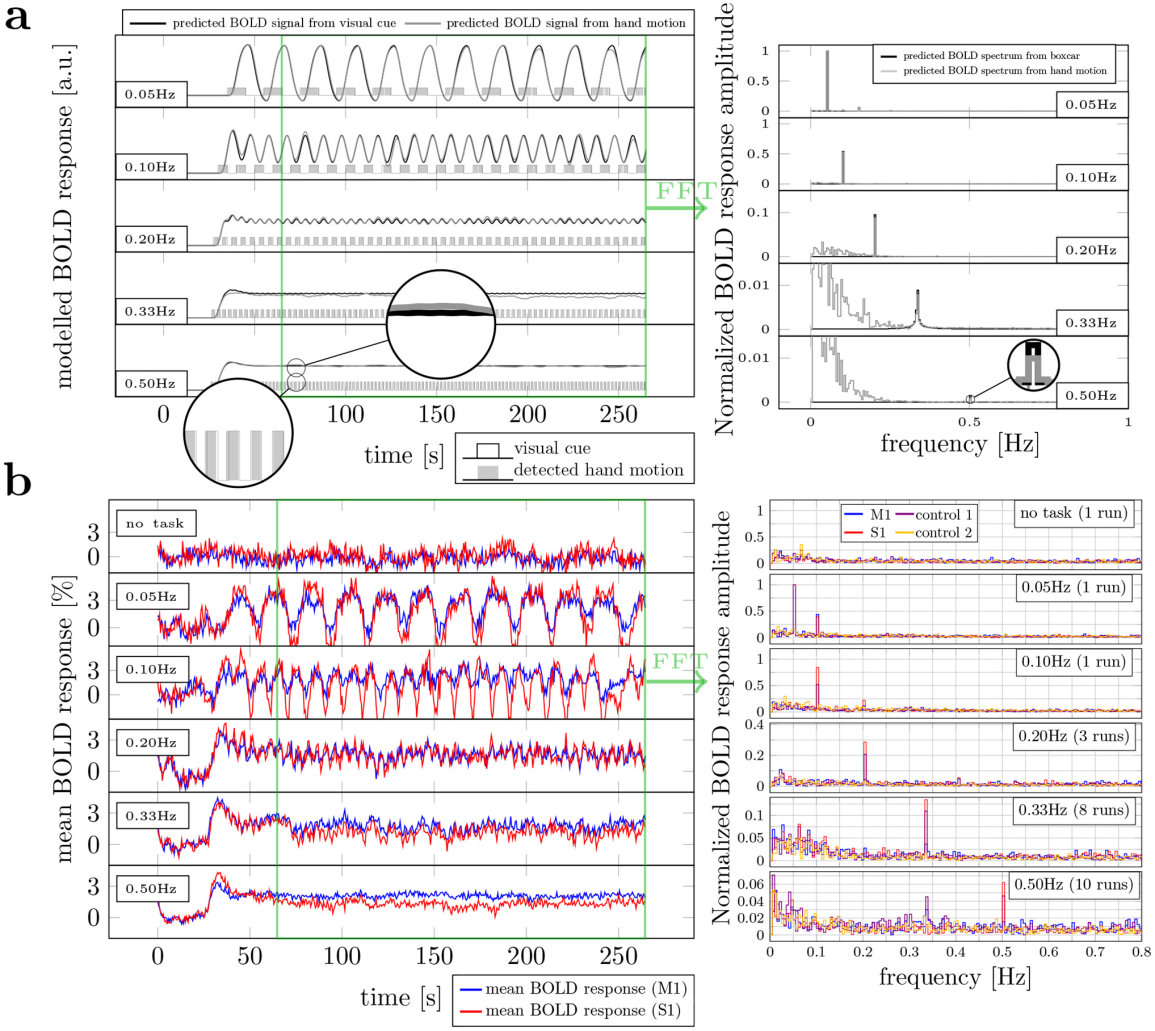
Experimental paradigm, model predictions, and experimental results. (a) Left panel: canonical HRF predictions of the BOLD response based on the ideal stimulus timing (black) and on detected hand motion (filled with gray). The initial 65 s were discarded to remove the transient signal component from the frequency analysis. The difference between the ideal and actual task performance at 0.5 Hz is shown in the zoomed inset. Right panel: spectra corresponding to the steady-state BOLD responses shown on the left. Spectral amplitudes were normalized to those of the reference stimulus (0.05 Hz). The y-axis was normalized to the peak observed at the target frequency in the corresponding 0.05 Hz experiment. (b) Left panel: blue and red plots show mean BOLD responses (averaged over runs) measured in M1 and S1 ROIs, respectively, in one representative subject. Right panel: corresponding spectra of these steady-state BOLD responses. In addition to M1 and S1 spectra, spectra from control ROIs are also plotted, in which no oscillations were observed. The spectral amplitudes from control ROIs were normalized to those of the reference responses, that is, the responses to 0.05-Hz stimulation within M1.

A series of fMRI experiments was performed to evoke BOLD oscillations in cortical areas S1 and M1 at stimulus frequencies up to 0.50 Hz. As shown on the left inFigure 1a, after a brief initial transient period, the expected BOLD response reaches a steady state. At higher frequencies, the steady-state BOLD response reaches a plateau with small oscillations around this new baseline. As expected, the amplitude of these oscillations becomes vanishingly small at high stimulus frequencies, highlighting the low-pass filter-like effect of the hemodynamic response. In particular, relative to the reference stimulus (0.05 Hz), the predicted BOLD responses to a 0.5-Hz frequency stimulus based on the canonical HRF were almost three orders of magnitude smaller. Unlike the previous work byLewis et al. (2016)in which a sinusoidal visual stimulus was used to evoke activation in human visual cortex, the periodic box-car stimuli used here contain multiple harmonics. However, these harmonics are suppressed by the low-pass filter effect of the hemodynamic response (Fig. 1a).

Simulations based on actual hand motion recorded in the scanner showed that unavoidable variations in task execution reduced the expected BOLD response amplitude up to 24%, suggesting that it should be even more challenging to experimentally observe these high-frequency BOLD oscillations. Nevertheless, even with slight variations in task execution, the predicted BOLD frequency response remains narrow.

### 3.2 Steady-state BOLD response analysis

Before attempting cortical depth analysis, we first investigated the average BOLD response throughout the M1 and S1 ROIs. Notwithstanding the discouraging model predictions based on the canonical HRF, distinct peaks were observed in the amplitude spectra of our BOLD response measurements at all stimulus frequencies in both M1 and S1, but not in the control ROI (Fig. 1b). These effects were also clearly visible in eight of nine subjects.

**Fig. 2.**
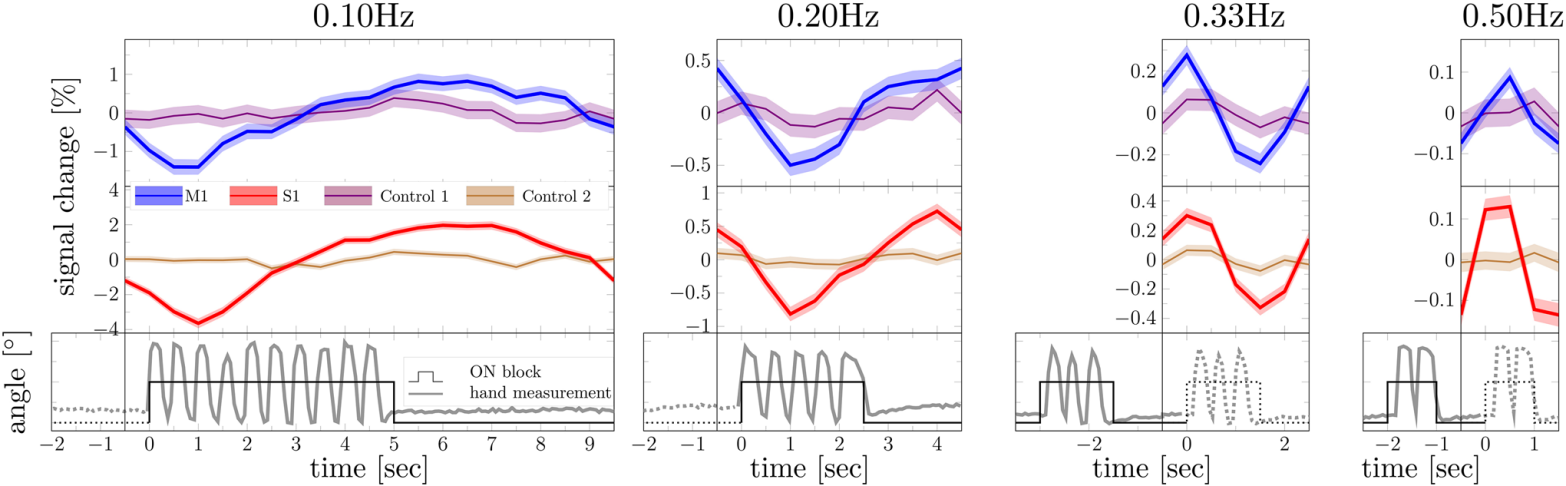
Mean trial-averaged responses and corresponding hand-motion recordings. The top and middle rows show mean trial-averaged responses from M1 (blue), control 1 (violet), S1 (red), and control 2 (brown) ROIs. The shaded areas represent the 95% confidence intervals. The bottom row shows the “on” block and recorded hand motion. All data shown here are from the same subject as shown inFigure 1. Note that, due to aliasing, positive peaks seen in 0.33-Hz and 0.5-Hz responses correspond to the stimulus in the preceding trial (solid line).

Figure 2shows the trial-averaged response (same subject as shown inFig. 1b).

Unlike our Fourier analysis presented inFigure 1, which isolates the sinusoidal component at the target (or fundamental) frequency, the trial-averaged response analysis considers the combined effect of all frequency components in the measured responses including the harmonics. At low stimulus frequencies, the BOLD response appears to be slightly asymmetric in time, with a duty cycle greater than 50%, indicating the presence of higher order harmonics in the response. As the stimulus frequency increases, the peak-to-peak BOLD amplitude becomes smaller, and the trial response shape becomes more symmetric (closer to 50% duty cycle). At 0.5 Hz, a clear sinusoidal response was observed in M1, with a 0.2% (0.4% in S1) peak-to-peak amplitude. Consistent with the results of our Fourier-based analysis, a 10-fold reduction in amplitude was observed between the BOLD response to stimulus frequencies 0.1- and 0.5-Hz stimulation. This trend was consistently observed in all subjects (Supplementary Fig. 7). Note that, the positive peaks seen in the 0.33-Hz and 0.5-Hz experiments appear to precede the stimulus onset, while in fact they correspond to the stimulus in the preceding trial (solid line in the bottom row ofFig. 2).

**Fig. 3.**
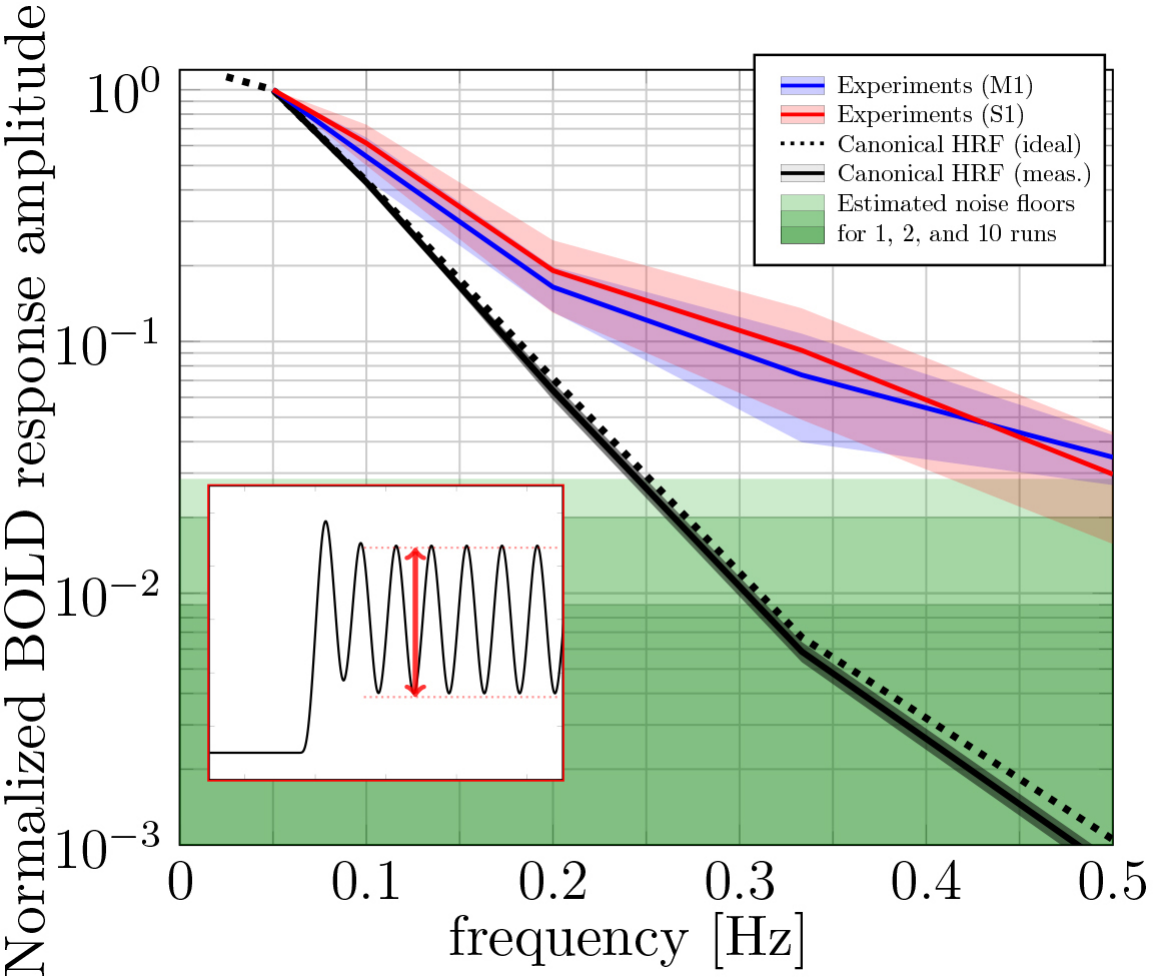
BOLD response amplitudes as a function of experimental paradigm frequency, averaged over whole M1 and S1 ROIs. The dotted and solid black lines show the canonical HRF predictions based on visual cues (i.e., the ideal stimulus timings) and hand motion recordings, respectively. The response amplitudes were normalized to those obtained using the 0.05-Hz stimulus. The shaded black area represents standard deviation across subjects. The blue and red lines show BOLD response amplitudes from M1 and S1, respectively. The response amplitudes were calculated subject by subject and each normalized to their respective 0.05-Hz measurement, then averaged over subjects. The blue and red shaded areas represent the standard deviations across subjects. The shaded green areas show the mean estimated noise floors for 1-, 2-, and 10-run averages. The small white subpanel illustrates how the steady-state BOLD oscillation amplitude was defined.

Figure 3shows the canonical HRF model predictions and the averaged spectral amplitudes from M1 and S1 across subjects normalized to those recorded at 0.05-Hz stimulation. Because spectral amplitudes were found to scale with percentage signal change, these points represent the fraction of the percentage signal change observed at a given frequency, relative to the percentage signal change observed at 0.05 Hz. Our data show the canonical model significantly underestimated the measured BOLD responses at all stimulus frequencies above 0.10 Hz. On average, the relative percentage signal changes in M1 (S1) were 1.3 (1.4), 2.3 (2.7), 11.0 (13.8), and 33.0 (28.2) times larger than model predictions of responses to 0.1, 0.2, 0.33, and 0.5 Hz stimuli, respectively. Note that noise estimates obtained from no-task experiments indicated that, based on the canonical HRF, the expected BOLD responses to 0.33- and 0.5-Hz stimulation would be expected to have fallen well below the noise floor (shaded green area).

### 3.3 Cortical depth-dependent analysis

**Fig. 4.**
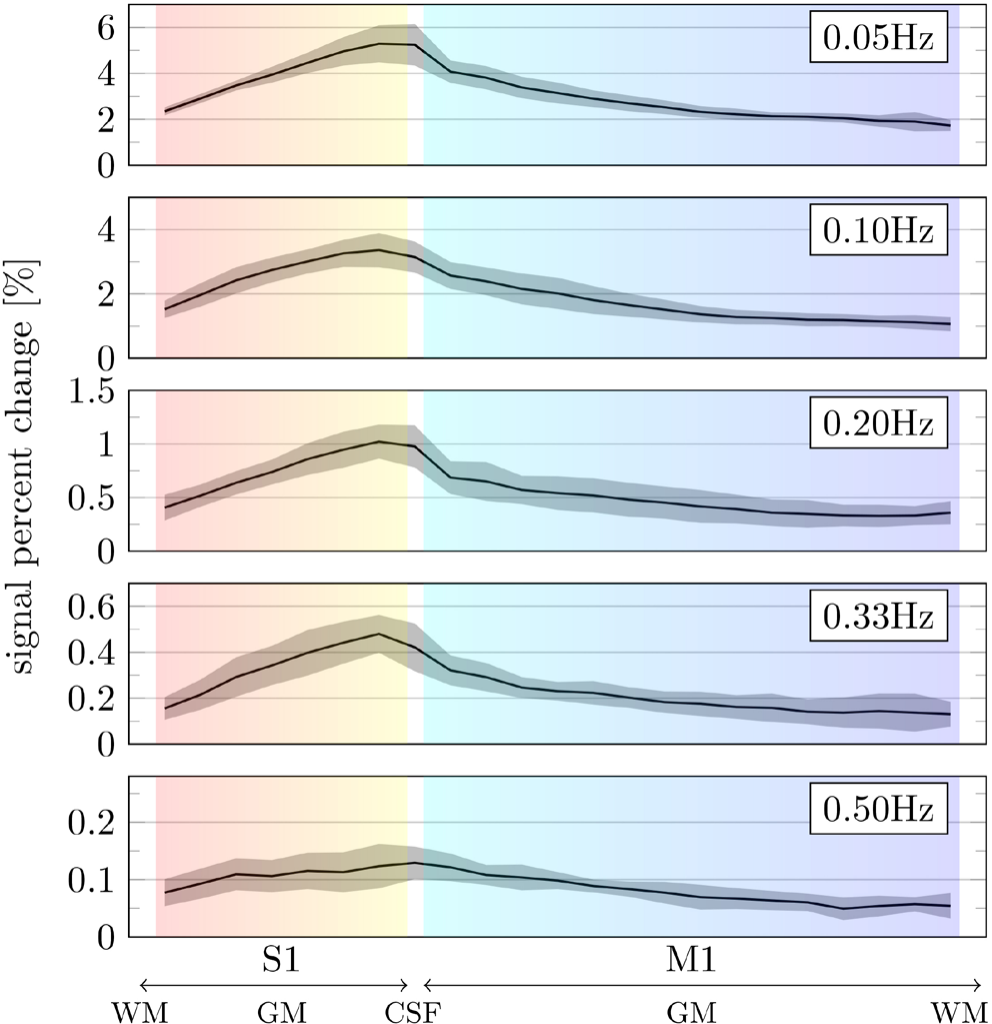
Cortical depth profiles of BOLD response amplitude as a function of stimulus frequency. Each panel shows the cortical depth profile of the BOLD response amplitude for stimuli of different frequency. Mean across subjects and the standard deviation across subjects are plotted as solid lines and shaded areas, respectively. Noise contribution to the 0.50-Hz experiment was analyzed further (seeSupplementary Fig. 9). Normalized depth is displayed from the S1 white matter interface through the pial surfaces (crossing the CSF) to the M1 white matter interface. S1 cortical depth is represented by the background color scale from red to yellow toward the pial surface. M1 cortical depth is represented by the background color scale from cyan to dark blue toward the white matter interface. (The ROIs are shown inSupplementary Fig. 6.)

To investigate whether the high-frequency BOLD responses change systematically across the cortical vascular hierarchy, where the smallest vessels are found within the tissue parenchyma and large draining veins lie on the cortical surface, a cortical depth analysis was performed in M1 and S1. Trial-averaged responses were calculated at each cortical depth and the percentage signal change was assessed as a function of cortical depth at each stimulus frequency (Fig. 4). As expected, stimuli up to 0.33 Hz produce cortical depth profiles in which the percentage signal change increases toward the pial surface, where we expect the BOLD signal to be dominated by the contribution from larger draining veins (Kim & Ugurbil, 2003;Polimeni et al., 2010;Turner, 2002;Zhao et al., 2004). In contrast, the 0.5-Hz experiment shows a much flatter response profile across cortical depths, indicating a relative reduction in the BOLD response near the pial surface. This suggests that large draining veins located near the pial surface contribute less to the BOLD response to high-frequency stimulation, as indicated in prior preliminary work (Gomez et al., 2024). Similar cortical depth profiles were observed using cortical depth-dependent spectral analysis (Supplementary Fig. 8).

**Fig. 5.**
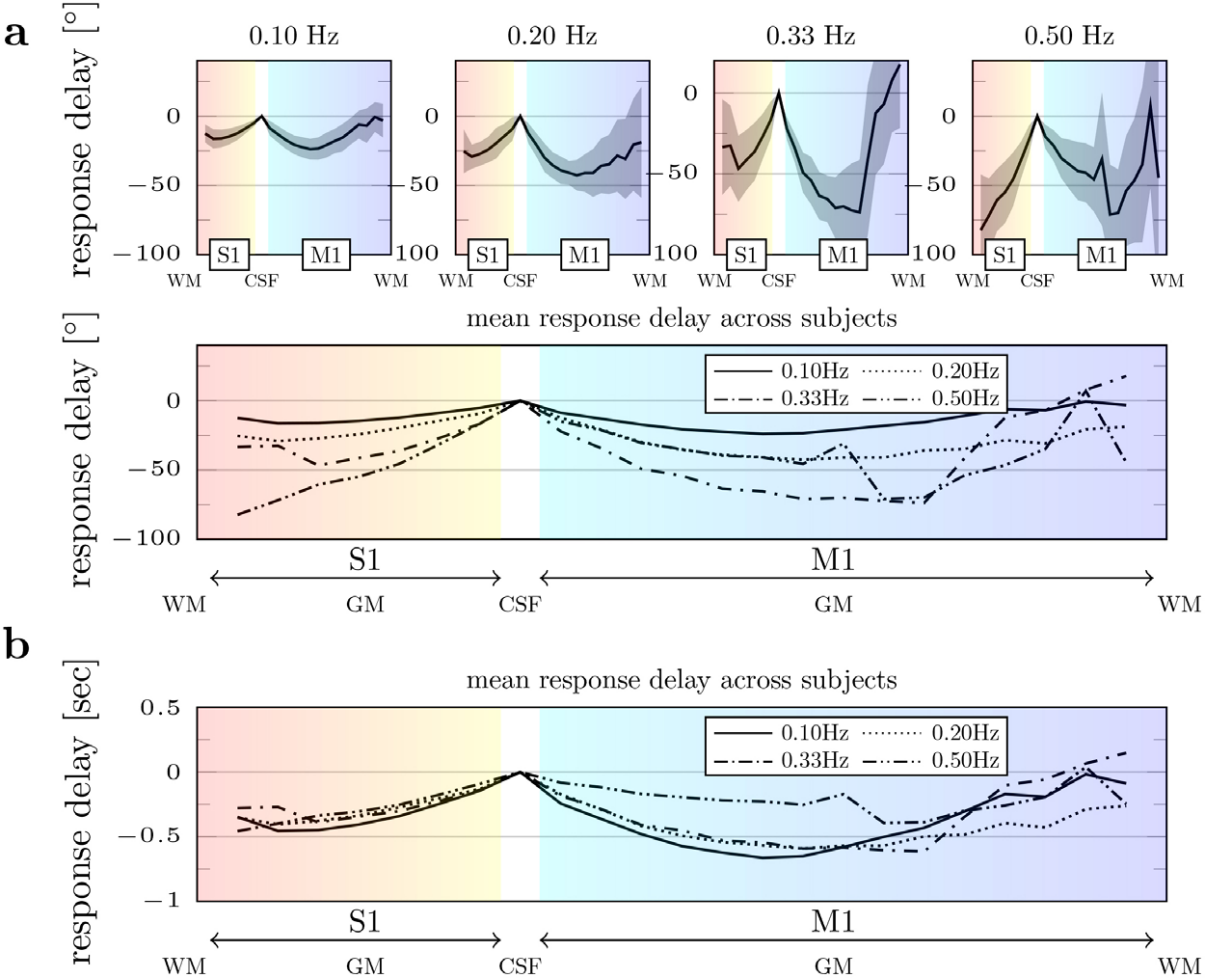
Cortical depth profile of the BOLD response delay time and the corresponding phase. Response delays were estimated from the cortical depth-dependent trial-averaged responses by fitting a sine function to the trial-averaged responses assuming the trial-averaged responses are sinusoidal. (a) The average response temporal phase plotted as a function of cortical depth. Even though the temporal delay remains similar for 0.05–0.33 Hz stimulation, the difference in phase delay between pial and parenchymal responses increases at higher stimulation frequencies. The goodness-of-fit between the sine function and the BOLD response is reported inSupplementary Figure 10. Standard deviations across all subjects are represented as shaded areas. Because there were only four time points before upsampling the 0.5-Hz stimulus frequency datasets, a higher variance can be seen in the profiles generated from the responses to the 0.50 Hz stimulus. (b) The relative time differences were normalized to the CSF ROI between M1 and S1. Note that the BOLD response delays plotted in panel (b) are relative to the CSF and are not easily related to the stimulus onset timing.

It is well established that, following stimulus onset, the supplying upstream arterial vessels dilate, which leads to a local increase in blood flow and a decreased deoxyhemoglobin concentration in the capillaries and venules (Hillman, 2014;Sirotin et al., 2009). Consequently, compliant downstream veins are thought to passively dilate or “balloon” to accommodate the increased cerebral blood flow. Therefore, we also analyzed trial-averaged response time delay and corresponding temporal phase across cortical depth (Fig. 5). On average, the response observed at the pial surface lagged behind deep S1 (~0.35 s) and middle layers of M1 (~0.50 s), suggesting that activity deep in S1 and M1 is out of sync by ~150 ms. These relative delays were comparable with observations made bySiero et al. (2011), who estimated a ~0.8-s transit time across cortical depths in M1.

Figure 5ashows the temporal phase of the BOLD oscillations across cortical depth. Although the temporal delay remains similar between the responses to 0.05–0.33 Hz stimuli (Fig. 5b), the phase difference between the pial surface and parenchyma increases substantially with stimulus frequency. Therefore, averaging all voxels across cortical depths, including out-of-phase pial and parenchymal responses, will lead to significant destructive interference in the mean BOLD response amplitude. To quantify the impact of this destructive interference, we compared the mean signal in the whole ROI with the mean of the absolute signal at each depth. In both M1 and S1, the cancellation of the BOLD response amplitudes due to the destructive interference increased with stimulus frequency as expected, although the losses were slightly different in the two areas (up to 21% attenuation in M1 and 12% attenuation in S1 in the responses to 0.5-Hz stimulation) (seeFig. 6).

**Fig. 6.**
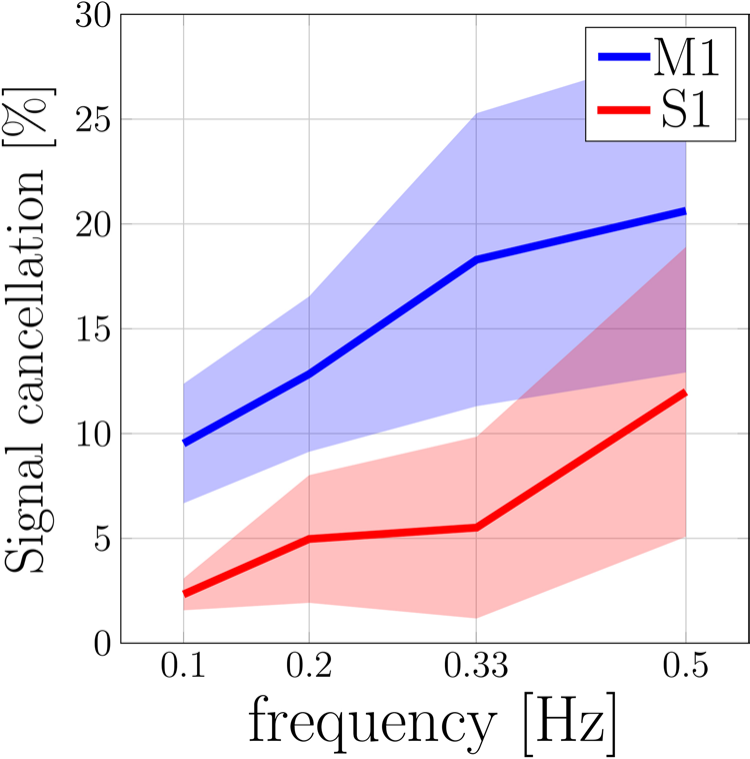
Signal cancellation due to destructive interference across cortical depths in M1 and S1. Signal cancellation was estimated by comparing the oscillating BOLD response amplitude resulting from averaging within cortical depths (Eq. [1]) and resulting from averaging within the entire ROI (Eq. [2]). In the ROI average, BOLD responses that are out-of-phase across depths cancel, leading to a smaller mean BOLD response amplitude. Averaging the oscillating BOLD response within individual cortical depths circumvents this destructive interference. The ratio between these was used to estimate the percentage amplitude loss caused by destructive interference (The standard deviation across subjects is represented by the shaded areas).

## 4 Discussion

Our results showed that the sensitivity to stimulus-driven high-frequency BOLD signal oscillations was similar in functionally and vascularly distinct cortical areas M1 and S1. Across cortical depth, we observed temporal shifts in the BOLD responses, presumably caused by different transit times within their drainage domains. As the stimulus frequency increased, these delays translated into increasingly large relative phase differences, most prominently visible in M1. When averaged across cortical depths, these phase differences caused the oscillating BOLD responses to interfere destructively. Moreover, we observed that, perhaps unexpectedly, the BOLD response amplitude close to the pial surface was observably lower at high stimulus frequencies, suggesting that the larger draining veins near the pial surface contributed less to the steady-state BOLD signal oscillations in response to high-frequency stimuli. In other words, our observations suggest that, in addition to low spatial specificity, large draining veins may also exhibit reduced temporal specificity.

### 4.1 Data quality

High-quality, high-SNR data are needed to study high-frequency stimulus-driven BOLD oscillations in the human brain. At 7 Tesla, between 8 and 10 4.5-min-long fMRI scans were needed to reliably measure 0.5-Hz oscillations in individual subjects (Fig. 3). In our experiments, an MRI-compatible dataglove was used to measure task performance. The recorded hand motion revealed some temporal variation, which reduced the predicted BOLD response amplitude, but did not lead to substantial changes in the predicted (Fig. 1a) and measured (Fig. 1b) BOLD signals.

Our experiments targeted 2-Hz finger flexion, not only because this is known to produce a strong BOLD response, but also because the BOLD response amplitude is expected to show little variation to small frequency deviations around 2 Hz (Siero et al., 2013). Recorded hand motion (Fig. 2, bottom row) clearly shows 10, 5, 3, and 2 peaks in the 0.10-, 0.20-, 0.33-, and 0.50-Hz stimulus response time courses, indicating a steady 2 ± 0.08 Hz rhythm. Therefore, it is unlikely that the 28-fold larger-than-predicted BOLD response was induced by variations in flexion frequency. Beside task performance, variations in attention and intention can also modulate BOLD response amplitude (Kanwisher & Wojciulik, 2000;Lau et al., 2004). Subjects generally reported that they found tasks with shorter “on” blocks to be more challenging, which could have led to increased attention. However, this is also unlikely to have altered the response amplitude to the extent observed.

The use of control ROIs was crucial in this study to rule out potential effects of stimulus-correlated motion because direct analysis of the head motion estimates provided little insight (Supplementary Fig. 11). Head motion detected during fMRI data preprocessing, including stimulus-correlated motion, is corrected by rigid realignment of the fMRI data. The question is: If we did not detect any motion, can we be sure it was not there? And, conversely, if we do detect motion, how do we know it has been sufficiently corrected? Examining intensity changes in control ROIs provides a more concrete and direct assessment of the effects of stimulus-correlated motion. The main results consist of data acquired in eight subjects, which only showed nonperiodic intensity fluctuations (<0.08% amplitude) in the control ROI, suggesting motion-related signal variations were minimal or absent (Supplementary Fig. 7). However, data from a ninth subject were excluded because we observed arm motion occurring between “on” and “off” blocks such that, even after image-based head motion correction, stimulus-correlated intensity variations were detected within the control ROI (Supplementary Fig. 12).

### 4.2 High-frequency stimulus-driven BOLD oscillations throughout the brain

Previous studies, using long ISIs, have also reported a disproportionately large BOLD response (~4–5-fold) to short-duration stimuli (Birn et al., 2001;Birn & Bandettini, 2005;de Zwart et al., 2009;Glover, 1999;Hirano et al., 2011;Janz et al., 2001;Liu & Gao, 2000;Miller et al., 2001;Nangini et al., 2005;Pfeuffer et al., 2003;Robson et al., 1998;Soltysik et al., 2004;Vazquez & Noll, 1998;Wager et al., 2005;Yeşilyurt et al., 2008). Although these short stimuli contain high-frequency components, it does not imply that high-frequency BOLD oscillations will be observable. When short ISIs are used, a steady state emerges, containing much smaller, yet detectable, BOLD oscillations. As first pointed out byLewis et al. (2016)in V1, these stimulus-driven BOLD oscillations were much larger than what was expected based on the canonical HRF (~30 fold in our case). Because the BOLD signal is, at least in part, shaped by the underlying vascular architecture, we set out to see whether high-frequency BOLD oscillations could also be detected in other cytoarchitecturally and vascularly distinct cortical regions S1 and M1. To our surprise, frequencies up to 0.5 Hz were not only detectible and much larger than predicted by the canonical HRF, both cortical areas showed similar responses as a function of frequency (Fig. 3).

The assumption that the neuronal activation exactly matches the stimulus timing—which in our case is a simple block-design paradigm consisting of a boxcar waveform—is an oversimplification that may lead to a mis-estimation of the true BOLD HRF. The voxel-level neuronal response is far more complex, and perhaps better modeled by an appropriate balance of excitatory and inhibitory signal components appropriate to the stimulus configuration and timing (Buxton et al., 2004;Havlicek et al., 2017). To account for whether more plausible neural dynamics may explain the discrepancy between prediction and observation, we replaced the boxcar waveform with a more realistic neural response waveforms reflecting one plausible choice for a balance between excitation and inhibition—with a strong response at stimulus onset followed by a reduced response reflecting adaptation—based on the neural response function proposed byBuxton et al. (2004), following the approach ofLewis et al. (2018). Even with a more plausible neuronal activity timing, a similarly large discrepancy between the modeled and observed BOLD responses remains (seeSupplementary Fig. 13).

More advanced, nonlinear, biophysically informed hemodynamic models, such as the Balloon model (Buxton, 2012;Buxton et al., 2004;Mildner et al., 2001) and delayed compliance models (Mandeville et al., 1999), may be able to provide a better match between prediction and observation. Although these flexible models can generate plausible BOLD responses, it can be difficult to set the model parameter values for a specific stimulus configuration given the known nonlinearities mentioned above, and it can also be difficult to interpret the model parameters when they are fitted to the fMRI data. Left unconstrained, a suitable fit in such a high-dimensional space does not guarantee convergence to physiologically relevant parameter values (Novikov et al., 2018). Yet, reasonable constraints on these parameter values based on empirical measurements from the literature could aid the convergence, however, these are often derived from responses to tasks with more conventional long ISIs and may not generalize to paradigms with short ISIs or durations. Therefore, the ability of these models to explain the origin of these fast hemodynamics is likely limited.

Although previous studies have revealed timing differences in the HRF between cortical areas (Handwerker et al., 2004), our measurements showed similar relative response amplitudes as a function of frequency in both M1 and S1 (Fig. 3). At the first glance, this suggests that, with our spatiotemporal resolution, their fine-scale differences in functional and vascular architecture (Duvernoy et al., 1981;L. Huber et al., 2017;Y. Yu et al., 2019) have little impact on the detectability of high-frequency stimulus-driven BOLD responses, at least when the responses are averaged over reasonably sized cortical ROIs. However, it is important to note that M1 and S1 can share some vascular infrastructure at the pial surface. These effects likely decrease deeper into the cortex (further away from the pial surface). Typically, S1 and M1 are approximately 2 mm and 4 mm thick, respectively. Therefore, even at 1.1 mm resolution, voxels deep in S1 and M1 are likely at least more than one voxel apart. The fact that we were still able to detect high-frequency stimulus-driven BOLD oscillations in the deeper cortical depths in both M1 and S1 (Figs. 4and5) thus provides additional support for this claim. Nevertheless, it would be worthwhile to examine the depth-dependent effect in more detail in a future study with smaller voxels, perhaps using a more powerful MRI system to obtain high-resolution data with less partial volume effects (Feinberg et al., 2023). Alternatively, future studies could validate this further by systematically studying responses across cortical depths of other cortical areas. Viewed alongside the work ofLewis et al. (2016), in V1, our work in M1/S1 suggests that high-frequency stimulus-driven BOLD oscillations may be observable in many cortical areas throughout the brain.

### 4.3 High-frequency BOLD oscillations across cortical depth

It has been shown previously that the temporal properties of the HRF vary systematically with cortical depths (Siero et al., 2011;Tian et al., 2010), which may also cause the temporal specificity of the BOLD response to change with cortical depth. In the BOLD responses to 0.5-Hz stimulation, shown inFigure 4, an increased attenuation in the BOLD oscillations was seen near pial surface. In the context of high-frequency stimuli, stimulus-driven hemodynamics in small parenchymal vessels close to the site of neural activation might behave differently from those in large veins at or near the pial surface. The amplitude and timing of stimulus-driven vascular responses depend on many factors.Gao et al. (2015)observed, in mice using low-frequency stimuli, that stimulus-driven dilation of venules and arterioles was greater toward the pial surface. They proposed that the structure of a perivascular funnel surrounding intracortical arterials and venules at the pial interface results in reduced mechanical restriction closer to the pial surface, which makes it easier for venules near the pial surface to expand (Pfannmoeller et al., 2021). However, at the same time, reduced elasticity of vessels near the pial surface that are not fully in contact with surrounding tissue can also prolong the time required for vascular diameter to return to baseline. Therefore, in the presence of high-frequency stimuli, changes in vessel diameter may be dampened resulting in a steady state in which the BOLD signal amplitude variations in draining veins are minimal. In mice,Drew et al. (2011)found that surface veins dilated in response to long-duration stimuli (>10 s) but dilations were negligible in response to short-duration stimuli. Perhaps these mechanical considerations become more relevant for rapid stimuli presented at 0.5 Hz? Alternatively, or in addition, mixing and cancellation of out-of-phase BOLD contributions collecting in larger draining veins might also explain our observation. The pial veins collect blood from different drainage pathways, leading to different transit times (Siero et al., 2011;Tian et al., 2010). Therefore, the multiplicity of different transit times may cause signal loss at the pial surface.

One of the hypotheses of this study was that increased spatial resolutions would aid the detection of high-frequency stimulus-driven BOLD oscillations. As shown inFigure 6, stronger signal cancellation effects indeed emerged with high-frequency stimuli. As the stimulus frequency increases, the same absolute time delay causes a larger relative phase difference. When signals from multiple depths are averaged, any out-of-phase responses will interfere destructively. This process is different from the integration of out-of-phase contributions from multiple parenchymal BOLD responses into large draining veins discussed earlier. Interestingly, stronger signal cancellations were seen in M1 than in S1 (Fig. 6). Perhaps this was due to the larger thickness of M1, accommodating more voxels, increasing our ability to detect signal cancellation effects, or perhaps due to the integration of BOLD responses with a broader range of relative phase differences due to the larger volume of tissue and longer transit time of blood drained from the parenchyma to the pial veins. In general, it is expected that both destructive interference effect and vascular responsiveness (discussed earlier) contribute to nonlinear response of the stimulus-driven BOLD response as function of frequency.

A central goal of cortical depth-dependent fMRI is to study layer-specific computations in the human cortex (Finn et al., 2021;Self et al., 2019;Stephan et al., 2019). In our experiments, neuronal activity was elicited in M1 and S1 by means of a 2-Hz finger flexion task. In S1, bottom-up feedforward signals (sensation, induced by friction of the fingertips inside the dataglove) first activate cortical layer IV, while top-down feedback signals (prediction) are believed to activate layers II/III and V/VI (Constantinople & Bruno, 2013;Thomson & Lamy, 2007). The temporal delay between neuronal activity related to sensation and prediction is expected to be small (on the order of 100 ms) and most likely overshadowed in our BOLD fMRI data by hemodynamic effects. In M1, our task generates cortical/thalamic input to layer II/III and spinal/thalamic output from layer V/VI, and potential recurrent activity in the deep layers, in addition to preparatory activity entering in superficial layers (Mao et al., 2011;Papale & Hooks, 2018;Weiler et al., 2008). Cortical/thalamic input from proprioception may be lagging ~100 ms behind the spinal/thalamic output to the muscles. Again, with our paradigm, it is difficult to separate these delays in neuronal activity from hemodynamic effects associated with drainage toward the pial surface. However, recurrent activity in the deeper layers may also be delayed relative to the spinal/thalamic output in the middle layers. This temporal ordering of neuronal activity matches the trends seen in our BOLD data in which the responses in deeper layers are delayed relative to the deeper layers, although we observe a delay of approximately 0.5 s that is likely far too slow to be purely driven by neuronal effects. Still, it offers a tantalizing explanation for the delayed signals observed near the white matter surface (Fig. 5). Alternatively, and equally tantalizing, the same observation could also be explained by intracortical vasculature draining blood toward white matter—looking carefully at the vascular anatomical data presented byDuvernoy et al. (1981), some “hook-shaped” drainage veins in the cortical gray matter can be seen moving down into the white matter and then reversing direction and traveling back into the gray matter. In addition, there is a drainage network near the white matter surface that could play a role. Interestingly the delayed BOLD responses deep in M1 were observed at all stimulus frequencies, which means it should be possible to study these dynamics using low-frequency paradigms where the contrast-to-noise ratio is higher and acquisition techniques with increased microvascular specificity, such as SE-BOLD and VASO, can be used more easily (Han et al., 2021;L. Huber et al., 2017;Y. Yu et al., 2019).

A combined in vivo microscopy and fMRI study in small animals conducted byTian et al. (2010)demonstrated that the middle cortical layers exhibited the earliest hemodynamic response onset times followed by the superficial cortical layers, however, the deep layers were not imaged. Our measured trend in response delays is consistent with these prior observations (Fig. 5). However, measurements deep in M1 deviate from this linear trend, an effect that was also seen byX. Yu et al. (2014)using rapid line-scanning fMRI, and perhaps also visible in the response onset time measured using two-photon microscopy (Uhlirova et al., 2016;Fig. 1supplement 1), suggesting a similar “U-shaped” cortical depth profile to what we observe. However, it is important to remember that these studies measured transient dynamics elicited by short-duration stimuli followed by a long ISI, not steady-state BOLD oscillations.

One of the limitations in our study is the imaging resolution. It is likely that the voxel size used in this study resulted in partial volume effects that caused a blurring in the cortical depth profiles, especially in S1 where the expected cortical thickness is around 2 mm. The 1.1 mm isotropic resolution was a trade-off between spatial resolution, brain coverage, temporal resolution, functional contrast (i.e., TE), and other factors. In addition, we were concerned that smaller voxels might not yield enough sensitivity to detect fast BOLD signal oscillations within a reasonable scan time.

In summary, while there may be some neuronal effects across cortical layers that could partly explain the observed delays in the BOLD response across cortical depths, given the short time scales of the neuronal dynamics compared with the longer time scales of the BOLD dynamics, it seems more likely that the delays observed in this study were dominated by vascular effects.

### 4.4 Implications for fast fMRI

Not long after the introduction of fMRI, despite the practical challenges stemming from the available MRI scanner technology,Bandettini and Cox (2000)showed that finger tapping experiments with a 0.25-Hz block paradigm produced a faint but detectable BOLD response (less than 0.2% signal change). However, considering the sacrifice in spatial resolution and brain coverage needed to achieve the necessary temporal resolution with the available MRI hardware, they advocated the use of judiciously chosen low-frequency task paradigms to elicit clearly detectable BOLD responses. However, state-of-the-art acquisition/reconstruction techniques and hardware improvements now provide access to fMRI data with much higher resolution in time and space (Feinberg et al., 2023;Moeller et al., 2010;Winkler et al., 2018). Our analysis suggests that, SNR permitting, small-voxel acquisitions are helpful even when studying high-frequency stimulus-driven BOLD oscillations, because the extra spatial resolution can be leveraged to mitigate destructive interferences of BOLD signals that occur when pooling data across cortical depths.

In a broader context, the observation of high-frequency BOLD oscillations in single subjects suggests the possibility of a wider range of fMRI paradigms to study motor control and somatosensation. Nevertheless, one must keep in mind that, although these high-frequency BOLD oscillations may be ~30 times larger than predicted by the canonical model, reliable observations at 1.1-mm isotropic resolution still require at least eight or more runs (~30 min). Looking beyond motor tasks, this also suggests that reliable detection of high-frequency BOLD signal oscillations (e.g., 0.33 Hz) in resting-state data may be feasible, albeit challenging.

## 5 Conclusions

In summary, our study demonstrates that BOLD response oscillations at task frequencies up to 0.50 Hz are observable in both M1 and S1, even in single subjects. Despite the differences in functional and vascular architecture between M1 and S1, the average BOLD response showed a similar frequency dependence. Collectively, together with the work ofLewis et al. (2016)in visual cortex, our observations suggest that, using modern MRI technology, high-frequency stimulus-driven BOLD oscillations may be detectable throughout the human brain.

Cortical depth analysis of M1 and S1 revealed compelling evidence of destructive interference effects, in support of the predictions made byPolimeni and Lewis (2021). Therefore, small-voxel acquisitions are helpful even when studying high-frequency stimulus-driven BOLD oscillations, because the extra spatial resolution can be leveraged to mitigate destructive interferences of BOLD signals. Moreover, we observed that, at stimulation frequencies above 0.33 Hz, the relative contribution of larger draining veins near the pial surface was significantly attenuated. This suggests that, in addition to low spatial specificity, large draining veins may also exhibit reduced temporal specificity.

## Supplementary Material

Supplementary Material

Supplementary Material Movie 1

Supplementary Material Movie 2

Supplementary Material Movie 3

Supplementary Material Movie 4

## Data Availability

The data and code are available from the corresponding author upon reasonable request.
