# Supplementary material for: Detectability and cortical depth dependence of stimulus-driven high-frequency BOLD oscillations in the human primary somatosensory and motor cortex
Source: Imaging Neurosci (Camb). 2025 Jan 13;3:imag_a_00427. doi: 10.1162/imag_a_00427 (PMC12319743; doi:10.1162/imag_a_00427)
Supplement: Supplementary Material [file imag_a_00427-supp.pdf]

Supplementary Materials

Detectability and cortical depth dependence of stimulus-driven high-frequency BOLD oscillations in the human primary somatosensory and motor cortex

Shota Hodono[1,2], Jonathan R. Polimeni[3,4], David Reutens[2,5], Martijn A. Cloos[1,2,5]

[1]Donders Centre for Cognitive Neuroimaging, Donders Institute for Brain, Cognition and Behaviour, Radboud University, Kapittelweg 29, 6525 EN Nijmegen
[2]Centre for Advanced Imaging, The University of Queensland, Brisbane, QLD, 4072
[3]Athinoula A. Martinos Center for Biomedical Imaging, Department of Radiology, Harvard Medical School, Massachusetts General Hospital, Charlestown, MA, 02129
[4]Harvard–MIT Program in Health Sciences and Technology, MIT, Cambridge, MA, 02139
[5]ARC Training Centre for Innovation in Biomedical Imaging Technology (CIBIT), The University of Queensland, Brisbane, QLD, 4072

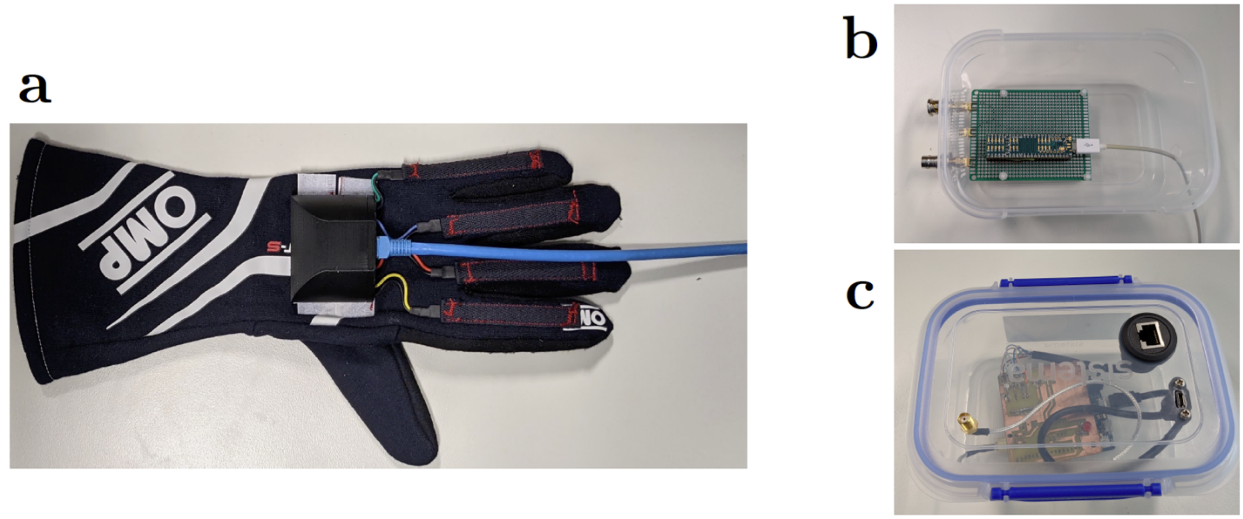

Supplementary Figure 1: **Photographs of custom-built dataglove.**

(a) Custom-built MR-compatible data glove. Subjects wore the dataglove during MRI scanning. The recorded hand motion kinematics data were encoded through an ethernet cable. (b) Trigger splitter. (c) Container holding the interface assembly. The container has three connections for receiving the trigger, receiving hand motion kinematics data, and sending the data to a computer for recording and storage.

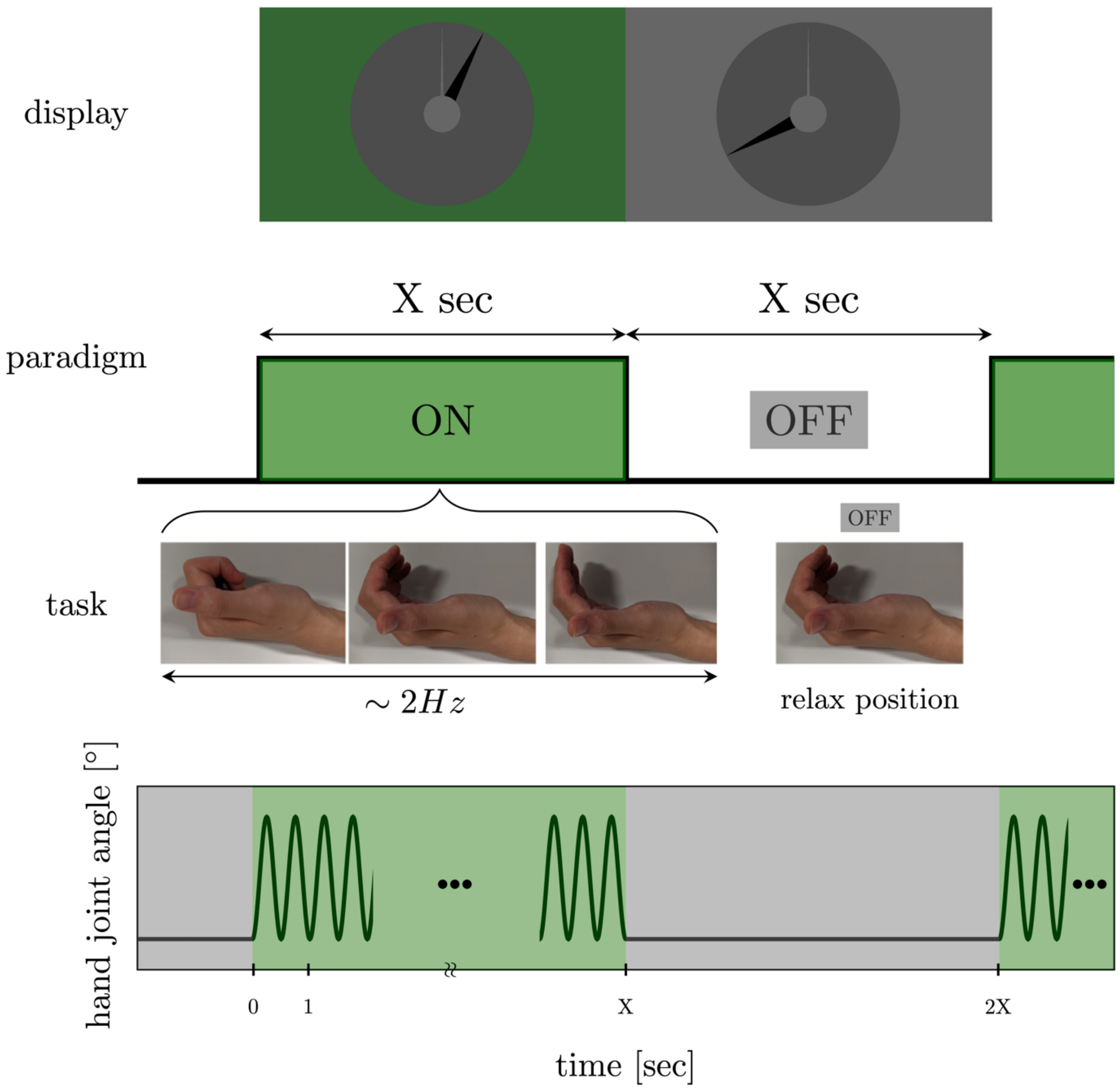

Supplementary Figure 2: **Illustration showing the visual cues used in our task paradigm.**
The duration of the 'on' and 'off' blocks (X) was varied to induce BOLD signal oscillations at different known frequencies. During 'on' blocks, the subject continuously performed a 2-Hz flexion task throughout the block. The 2-Hz rhythm was provided through visual cues. During 'off' blocks, subjects were asked to maintain a relaxed hand posture. To minimize B_0 changes and mechanical motion, subjects were asked to only move their fingers and not move their arm (resting in a comfortable position and stabilized using cushions). The bottom row shows the expected hand motion. Note that, during each fMRI experiment, the subjects wore our custom-built MR compatible dataglove to monitor these hand motions. The task can also be seen in Supplementary Movies 1–4.

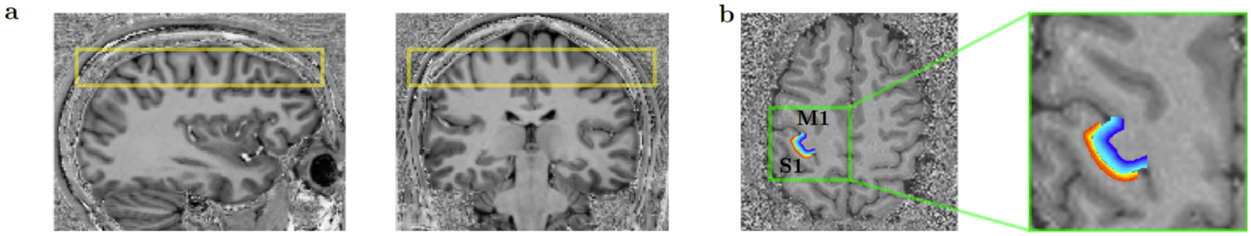

Supplementary Figure 3: **Brain coverage and cortical depth ROIs in S1 and M1.**
(a) The yellow box overlaid on an MP2RAGE image indicates the slice positioning for our fMRI experiments (18 slices). (b) The cortical depth ROIs in S1 (yellow to red) and M1 (cyan to blue) were computed using LayNii (Huber et al. 2021). The cortical depth ROIs are here shown overlaid on the T_1-EPI based synthetic MP2RAGE used as anatomical reference data that are distortion-matched to the fMRI data.

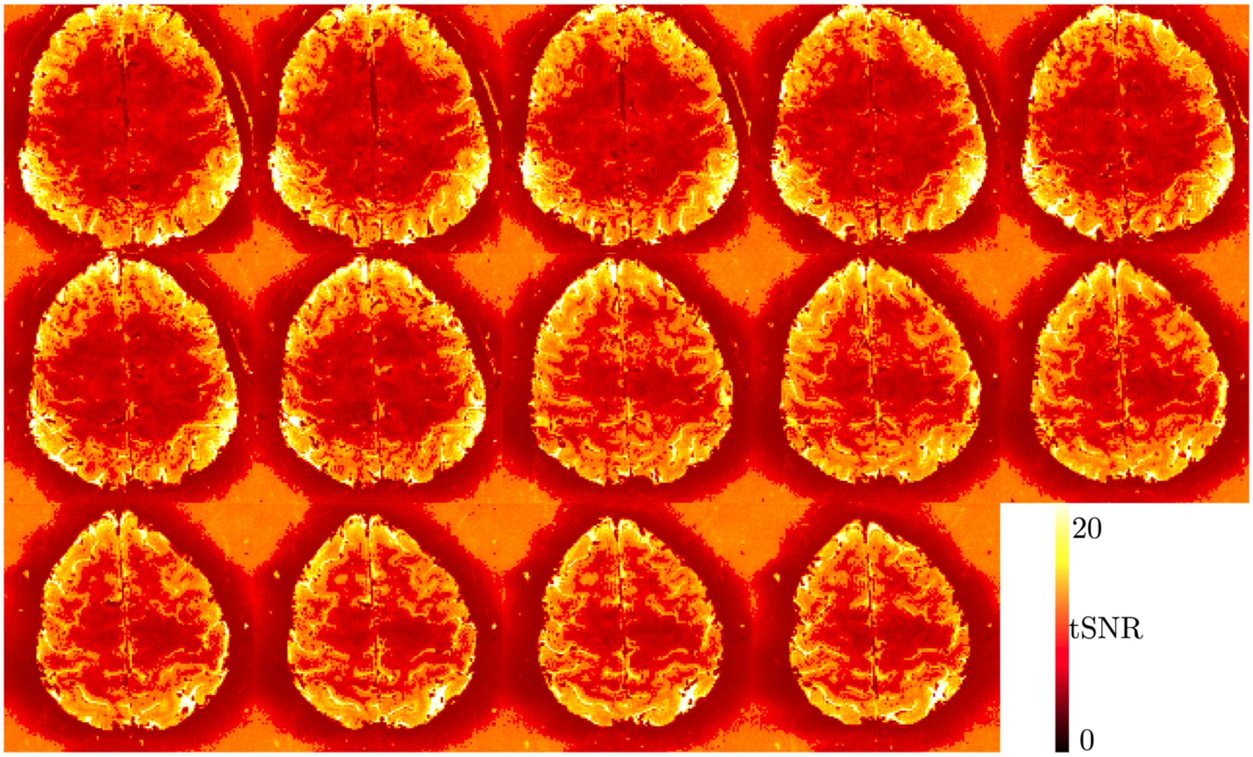

Supplementary Figure 4. **tSNR maps**. tSNR was computed from a single run without task (530 volumes) to inspect potential g-factor related noise amplifications. From top left to bottom right, tSNR maps of slice 3 to 16 were shown. No distinct g-factor penalties from multiband and in-plane acceleration were observed.

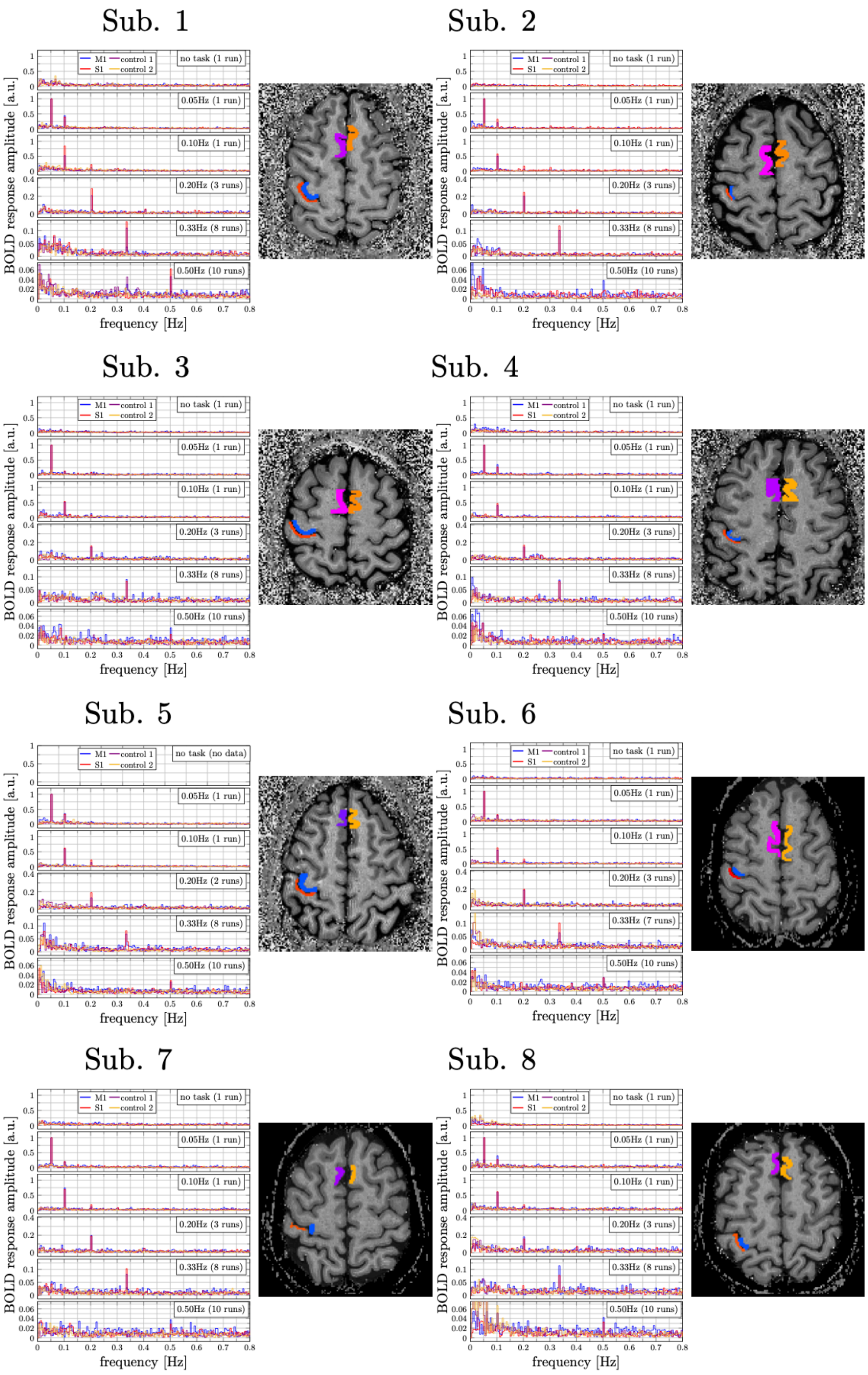

Supplementary Figure 5: **Frequency analysis for individual subjects.**

Spectra from four ROIs (M1, S1, control 1, and control 2) for each fMRI experiment across stimulus frequencies for all subjects and corresponding ROIs are shown. The spectral amplitudes from the control ROIs were normalized to the reference response, i.e., the response to the 0.05-Hz stimulation measured in M1.

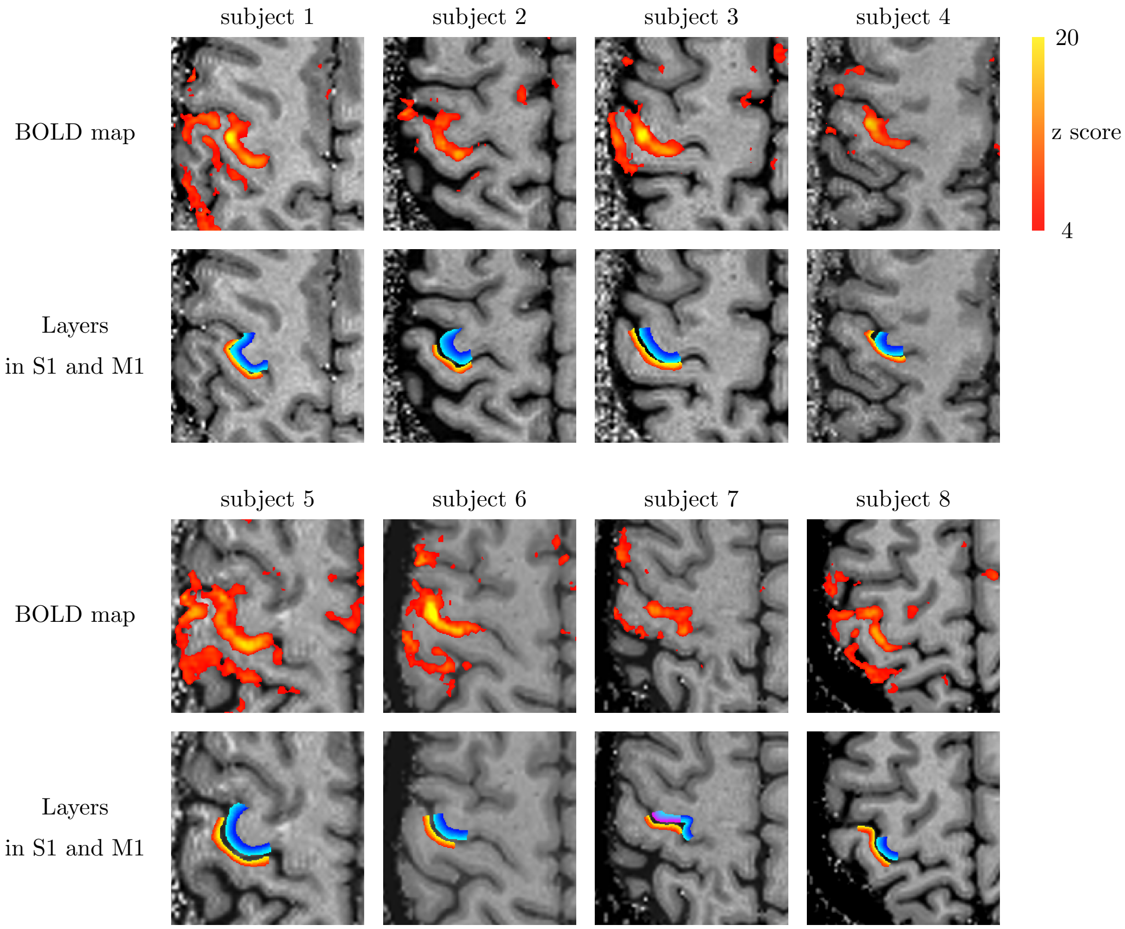

Supplementary Figure 6: **Activation maps from localizer experiment and cortical depth ROIs for all subjects.** The z-score maps from the functional localizer experiment and cortical depth ROIs were mapped into the synthesized MPRAGE image generated from T_1-weighted EPI data. In the upsampling process, no explicit smoothing was applied. The cortical depth ROIs in S1 (yellow to red) and M1 (cyan to blue) were computed using LayNii (Huber et al. 2021). In subject 7, the M1 ROI was drawn on the intersection between activation map and hand knob area. The separate ROI drawn in the purple-to-cyan colorscale was not included in the final M1 ROI because this region was far from the known hand-knob representation in M1 and the BOLD responses found in this separate ROI was far noisier than those of the other ROIs.

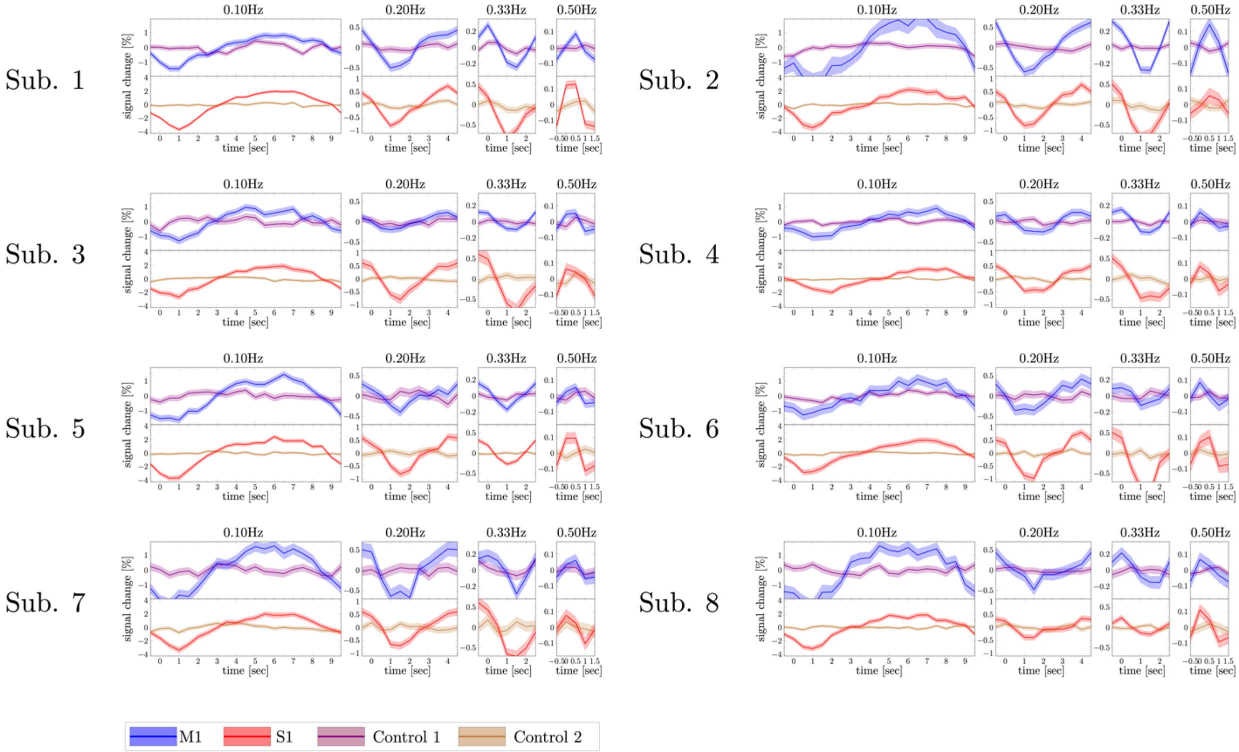

Supplementary Figure 7: **Trial-average responses for individual subjects.**

Trial-average response plots for individual subjects (Subjects 1–8) for all task frequencies up to 0.50 Hz. In all panels, the top and bottom rows show mean trial-average responses from M1 (blue), control 1 (violet), S1 (red), and control 2 (brown) ROIs. The shaded areas represent the 95% confidence intervals.

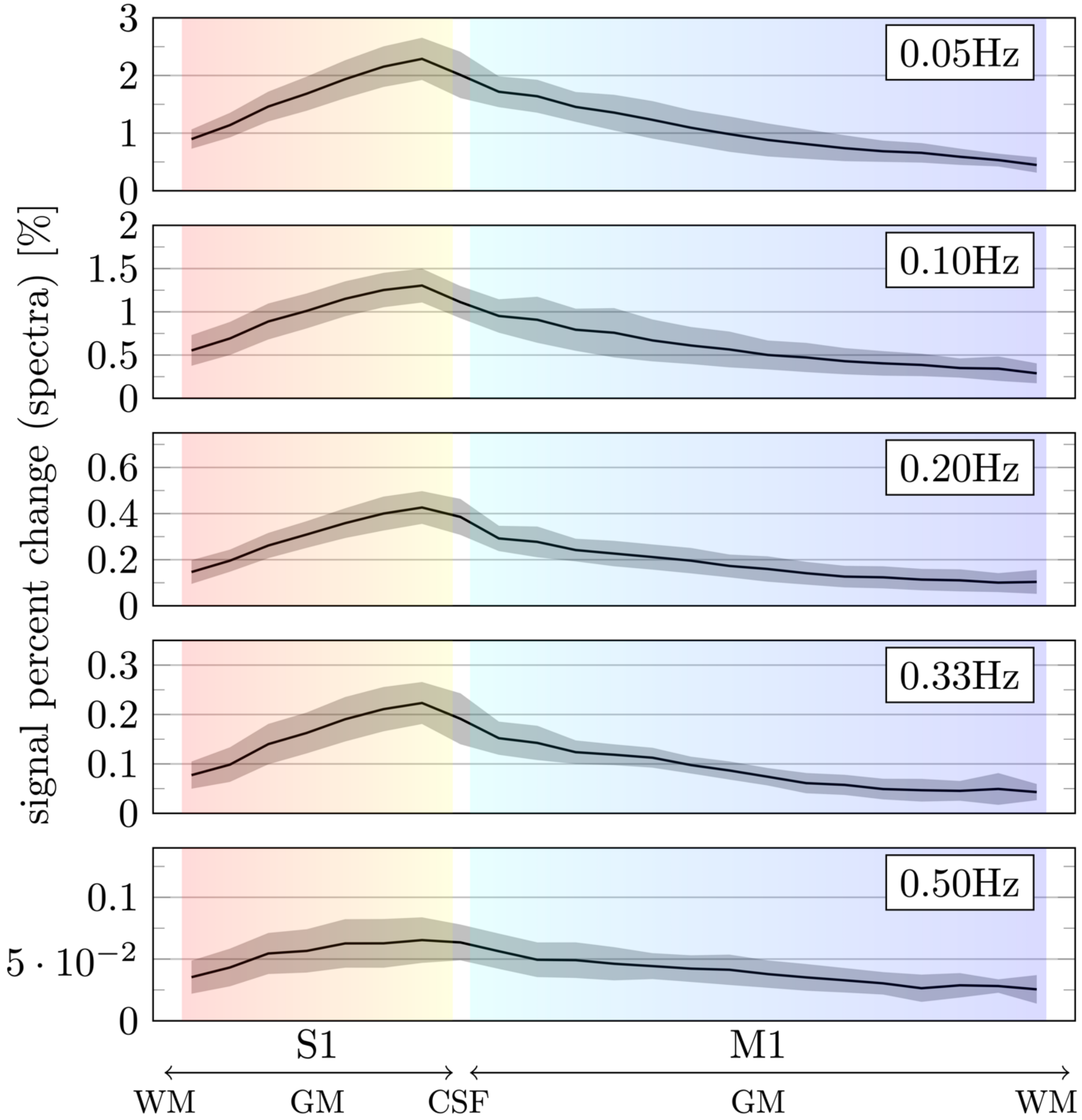

Supplementary Figure 8. **Cortical depth profiles from frequency analysis**. Frequency response at task frequency was divided by the DC component to estimate the signal percent change.

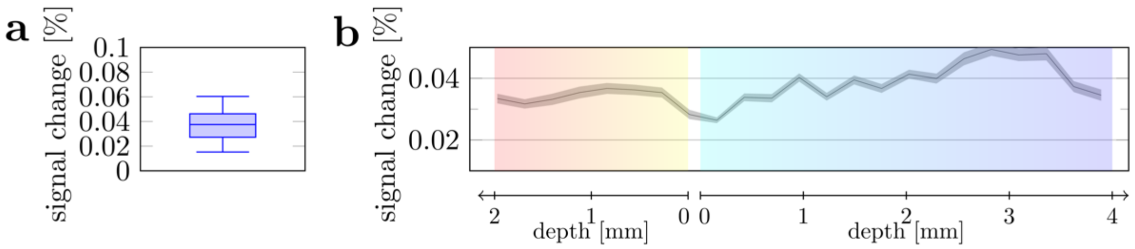

Supplementary Figure 9: **Cortical depth dependence of noise observed in experiments without a task.** (a) Box plot of expected noise floor for responses to 0.5-Hz across cortical depth (S1 and M1), computed using 10 runs without any task from a single subject. Since the BOLD signal in this dataset is not driven by any task, it is expected that the percent signal change represents only thermal and physiological noise contributions. The median value across cortical depth was 0.037%. (b) Percent signal change as a function of cortical depth using 5 out of 10 runs at 0.5 Hz (252 combinations). The black line represents the mean profile of 252 combinations. The shaded gray area represents the 95% confidence intervals across 252 combinations.

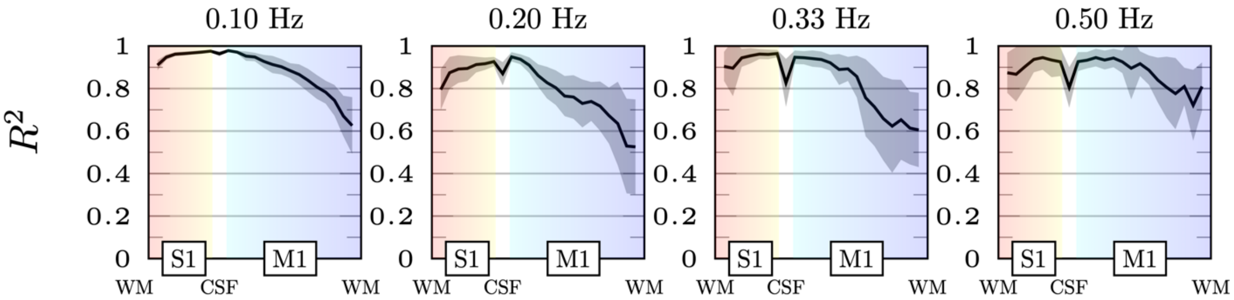

Supplementary Figure 10: **Goodness of fit of predicted BOLD response as a function of cortical depth across task frequencies**. Goodness of fit between upsampled trial-average response and a sine curve representing the presumed BOLD was estimated using R^2 values for each subject and depth. The means across the subjects are plotted as solid lines, and the shaded areas represent confidence intervals across subjects. Cortical depth is displayed from the S1 white matter interface through the pial surface (crossing the CSF) to the M1 white matter interface. S1 is highlighted in red to yellow (white matter to the pial surface). M1 is highlighted in cyan to dark blue (pial surface towards the white matter interface).

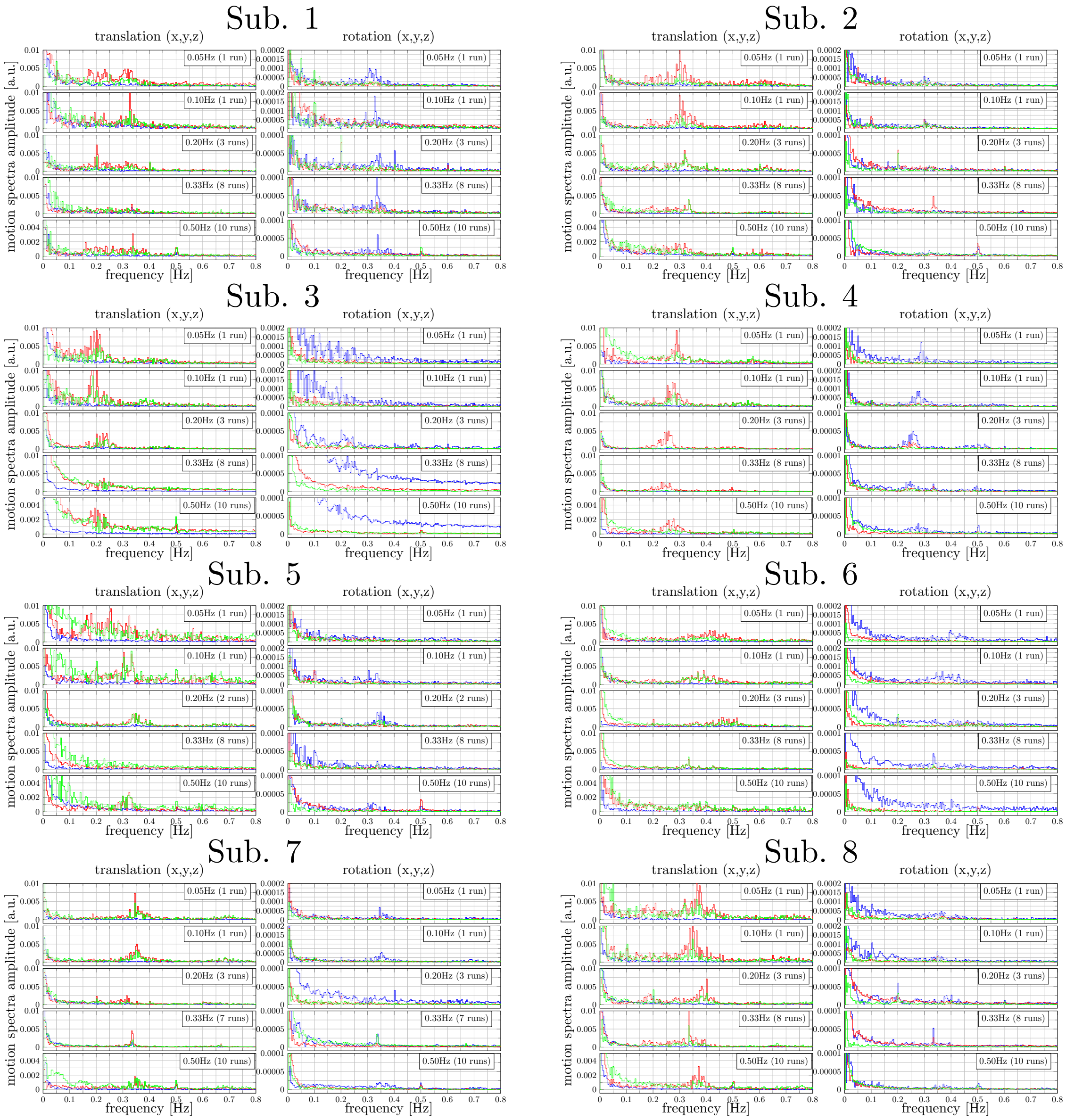

Supplementary Figure 11: **Frequency analysis of motion estimates.**

Estimated head motion during fMRI scans were analyzed in same manner as our frequency-domain analysis. The timeseries of the six motion parameters (translation in *x*, *y*, and *z* directions, and rotation about the *x*, *y*, and *z* axes) were Fourier transformed run by run then averaged in the frequency domain; the absolute values were plotted in blue, red, and green for *x*, *y*, and *z*, respectively. Since no reference spectral intensity is available, the normalization used on our fMRI frequency-domain analysis was not performed here.

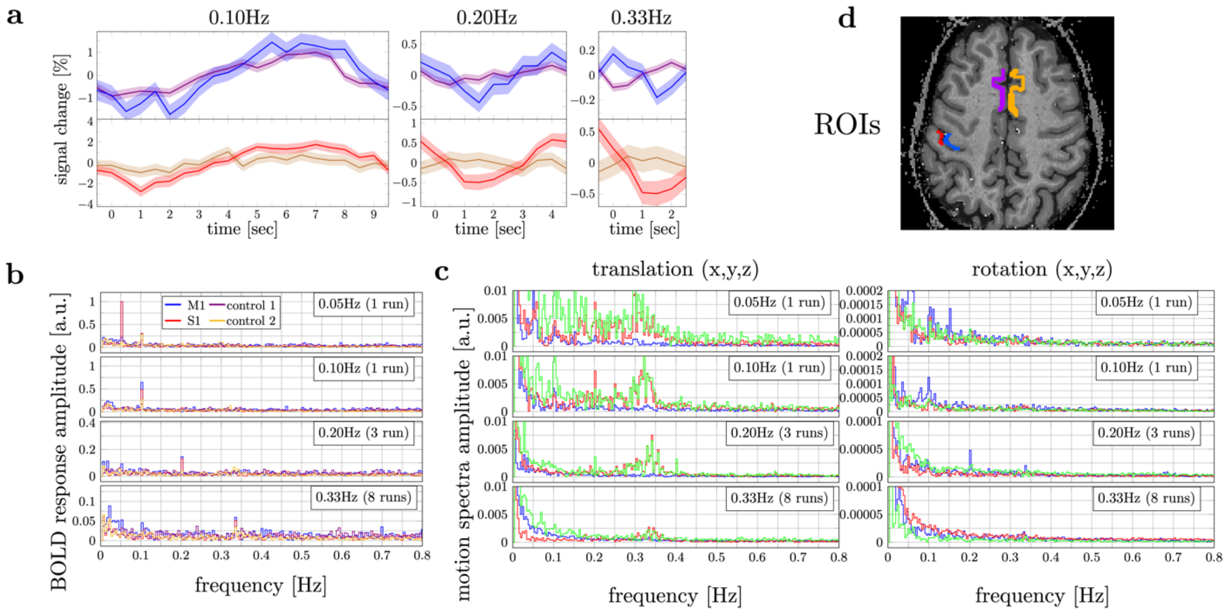

Supplementary Figure 12: **fMRI results and motion estimates from the ninth subject that was excluded from the analysis**. (a) Results from time-domain analysis, showing trial-average responses from M1 (blue), control 1 (violet), S1 (red), control 2 (brown) ROIs. The shaded areas represent the 95% confidence intervals. The bottom row shows the 'on' block and recorded hand motion. (b) Results from frequency-domain analysis, showing spectra from M1, S1, and control ROIs. (c) Results from frequency-domain analysis of motion parameter estimations. (d) Locations of all ROIs in this subject.

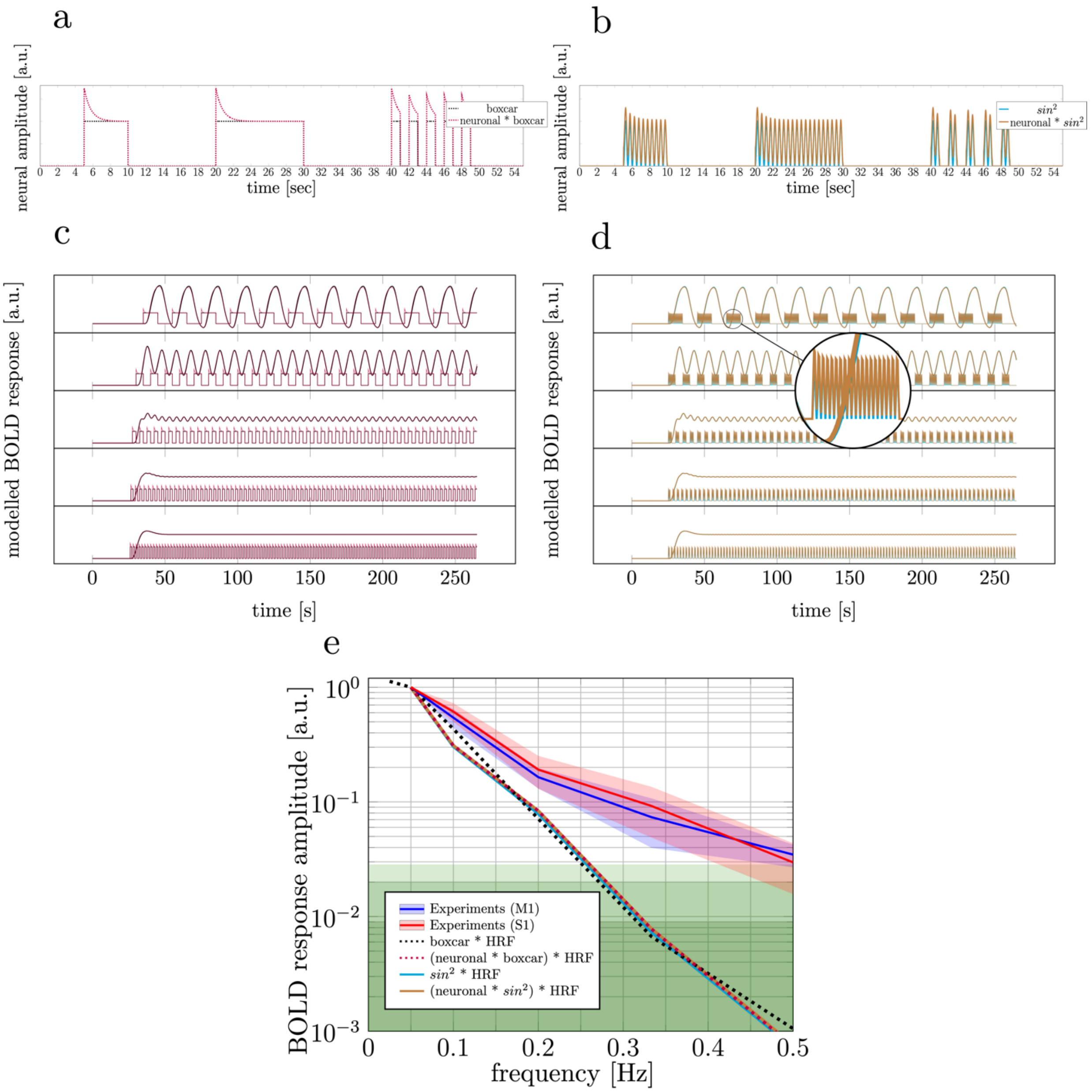

Supplementary Figure 13: **Predicted BOLD response amplitude using a realistic neuronal response.**
(a) An example of nonlinear neuronal responses derived from a boxcar input. (b) An example of nonlinear neural responses derived from a sine-squared input. (c) Predicted BOLD responses obtained by convolving the canonical HRF and boxcar (black) and nonlinear neural response based on the boxcar inputs (purple). (d) Predicted BOLD responses by convolution of canonical HRF and sine square (cyan) and nonlinear neural response based on a 2-Hz sine-squared input function, mimicking the finger flexing task during the 'on' block (brown). (e) Comparison of the experimentally observed BOLD response amplitudes as a function of stimulus frequency vs. simulated results using different nonlinear neural response. Here, several parameters (inhibitory gain factor: 0.0–3.0, inhibitory time constant: 1–3 s (Buxton et al. 2004) for the nonlinear neuronal responses were evaluated in the simulation, however all results underestimated the observed BOLD responses to the high-frequency stimuli.

Supplementary Table 1: **Details of experimental paradigm and the numbers of runs.**

Frequency	no task	0.025Hz	0.05Hz	0.10Hz	0.20Hz	0.33Hz	0.50Hz
On block duration	-	20	10	5	2.5	1.5	1.0
Off block duration	-	20	10	5	2.5	1.5	1.0
Hand	-	Left + Right	Left	Left	Left	Left	Left
Number of runs per subject	1	1	1	1	3	8	10

Supplementary Table 2: **Details of number of runs performed for each subject.** (*: All runs acquired for subject 5 had 490 measurements (without 20-s off block in the beginning), while all other runs acquired for other subjects had 530 measurements (with 20-s off block in the beginning).) Subject 9 was not invited for a second session because significant motion contributions were seen in dataset acquired in session 1 (see Supplementary Figure 8).

Frequency			0.05Hz	0.10Hz	0.20Hz	0.33Hz	0.50Hz	no task
number of runs	subject 1	session 1	1	1	3	8	-	1
		session 2	1	1	-	-	10	1
	subject 2	session 1	1	1	3	8	-	1
		session 2	1	1	-	-	10	1
	subject 3	session 1	1	1	3	8	-	1
		session 2	1	1	-	-	10	1
	subject 4	session 1	1	1	3	8	-	1
		session 2	1	1	-	-	10	1
		session 3	1	-	-	-	-	10
	subject 5*	session 1	1	1	2	-	-	-
		session 2	1	-	-	8	-	-
		session 3	1	1	-	-	10	-
	subject 6	session 1	1	1	3	7	-	0
		session 2	1	1	-	-	10	1
	subject 7	session 1	1	1	3	8	-	0
		session 2	1	1	-	-	10	1
	subject 8	session 1	1	1	3	-	-	-
		session 2	1	1	-	8	-	-
		session 3	1	1	-	-	10	1
	subject 9	session 1	1	1	3	8	-	-
		session 2	not invited					

Supplementary Table 3: **Numbers of voxels in S1 and M1 ROIs.**

		number of voxels S1	M1
subject 1	session 1	555	779
	session 2	643	968
subject 2	session 1	504	927
	session 2	347	775
subject 3	session 1	309	817
	session 2	410	742
subject 4	session 1	532	861
	session 2	349	580
subject 5*	session 1	426	827
	session 2	508	982
	session 3	433	976
subject 6	session 1	261	473
	session 2	256	426
subject 7	session 1	347	331
	session 2	346	386
subject 8	session 1	372	376
	session 2	339	345
	session 3	466	538
subject 9	session 1	210	258
	session 2	-	-

Supplementary Table 4: **Estimated cortical thickness in S1 and M1.**

		thickness [mm]	
		S1	M1
subject 1	session 1	1.44 ± 0.26	2.82 ± 0.35
	session 2	1.45 ± 0.30	2.83 ± 0.40
subject 2	session 1	1.83 ± 0.29	3.34 ± 0.44
	session 2	1.60 ± 0.26	3.31 ± 0.50
subject 3	session 1	1.62 ± 0.23	2.81 ± 0.34
	session 2	1.70 ± 0.24	2.98 ± 0.30
subject 4	session 1	1.46 ± 0.24	2.82 ± 0.29
	session 2	1.41 ± 0.28	2.83 ± 0.27
	session 3	1.46 ± 0.24	2.82 ± 0.29
subject 5*	session 1	1.67 ± 0.21	2.97 ± 0.45
	session 2	1.56 ± 0.26	2.97 ± 0.47
	session 3	1.47 ± 0.24	2.54 ± 0.36
subject 6	session 1	1.59 ±0.22	2.71 ± 0.27
	session 2	1.46 ± 0.20	2.77 ± 0.34
subject 7	session 1	1.44 ± 0.23	2.31 ± 0.30
	session 2	1.61 ± 0.25	2.39 ± 0.37
subject 8	session 1	1.63 ± 0.28	2.80 ± 0.30
	session 2	1.60 ± 0.25	2.77 ± 0.26
	session 3	1.72 ± 0.26	2.93 ± 0.46

Supplementary Movies 1–4: **Tasks performed at different frequencies.** The ethernet cable seen in Supplementary Figure 1 was disconnected for these movies.

References

Buxton, R. B., Uludağ, K., Dubowitz, D. J., & Liu, T. T. (2004). Modeling the hemodynamic response to brain activation. Neuroimage, 23, S220-S233. https://doi.org/10.1016/j.neuroimage.2004.07.013

Huber, L. R., Poser, B. A., Bandettini, P. A., Arora, K., Wagstyl, K., Cho, S., Goense, J., Nothnagel, N., Morgan, A.T., van den Hurk, J., Müller, A., Reynolds, R.C., Glen, D.R., Gobel, R., and Gulban, O. F. (2021). LayNii: A software suite for layer-fMRI. NeuroImage, 237, 118091. https://doi.org/10.1016/j.neuroimage.2021.118091
